# Multi-omics pan-cancer analyses identify MCM4 as a promising prognostic and diagnostic biomarker

**DOI:** 10.1038/s41598-024-57299-1

**Published:** 2024-03-18

**Authors:** Yanxing Li, Wentao Gao, Zhen Yang, Zhenwei Hu, Jianjun Li

**Affiliations:** 1 Xi’an Jiaotong University Health Science Center, Xi’an, 710000 Shaanxi People’s Republic of China; 2 Department of Cardiology, Jincheng People’s Hospital Affiliated to Changzhi Medical College, Jincheng, Shanxi People’s Republic of China

**Keywords:** MCM4, Multi-omics, Pan-cancer, Bioinformatics, Immune infiltration, Prognosis, Biomarker, Cancer genetics, Tumour biomarkers, Cancer, Computational biology and bioinformatics, Drug discovery, Immunology, Biomarkers

## Abstract

Minichromosome Maintenance Complex Component 4 (MCM4) is a vital component of the mini-chromosome maintenance complex family, crucial for initiating the replication of eukaryotic genomes. Recently, there has been a growing interest in investigating the significance of MCM4 in different types of cancer. Despite the existing research on this topic, a comprehensive analysis of MCM4 across various cancer types has been lacking. This study aims to bridge this knowledge gap by presenting a thorough pan-cancer analysis of MCM4, shedding light on its functional implications and potential clinical applications. The study utilized multi-omics samples from various databases. Bioinformatic tools were employed to explore the expression profiles, genetic alterations, phosphorylation states, immune cell infiltration patterns, immune subtypes, functional enrichment, disease prognosis, as well as the diagnostic potential of MCM4 and its responsiveness to drugs in a range of cancers. Our research demonstrates that MCM4 is closely associated with the oncogenesis, prognosis and diagnosis of various tumors and proposes that MCM4 may function as a potential biomarker in pan-cancer, providing a deeper understanding of its potential role in cancer development and treatment.

## Introduction

Cancer imposes tremendous suffering on individuals, and its complex pathogenesis remains an intricate challenge for medical professionals^1,2^. Despite advances in cancer treatments such as radiotherapy, chemotherapy, targeted therapy and immunotherapy, there is still much to be discovered about the underlying mechanisms that drive tumor development and progression^3,4^. Recent advancements in bioinformatic analyses have significantly transformed the landscape of cancer research, offering robust methodologies for the identification of emerging biomarkers^5^. Central to this endeavor is the adoption of pan-cancer analysis, a sophisticated approach facilitating comparative investigations of gene expression patterns across a spectrum of cancer types^6–8^. This approach enables the identification of shared and distinct features among different cancer types, providing valuable information for diagnostic, prognostic, and therapeutic purposes. The accessibility of extensive genomic datasets, including TCGA and GTEx, has greatly facilitated the analysis of pan-cancer studies^9,10^. These resources offer a wealth of data that can be mined to uncover crucial genetic markers and elucidate their roles in cancer development, ultimately paving the way for improved patient outcomes.

MCM4 protein is a family member of the MCM (minichromosome maintenance) family, which comprises six highly conserved proteins MCM2-7^11–13^. The preservation of the MCM2-7 complex at the microscopic level is crucial for the proper execution of replication helicase function, which is indispensable for preserving the integrity of the cell cycle^14–16^. A deficiency in MCM has been linked to detrimental consequences, including genome instability (GIN), a hallmark of cancer and developmental abnormalities in murine models^17^. MCM4 has been shown to tightly interact with MCM6 and MCM7 to form a functional complex^18,19^. Research has demonstrated that this complex possesses intrinsic DNA helicase activity, a vital enzymatic function that participates in a wide range of cellular processes including replication initiation, DNA repair, and recombination^18,20^. Increasing evidence has confirmed the overexpression of MCM4 in several cancers like hepatocellular carcinoma, soft-tissue sarcoma, esophageal cancer, glioma and uterine corpus endometrial carcinoma^21–26^. The overexpression of MCM4 may lead to aberrations in the cell cycle, promoting uncontrolled cell proliferation, thus accelerating tumor growth and metastasis. On the other hand, mutations in the MCM4 gene are also associated with the occurrence of cancer. Some studies have indicated the presence of MCM4 gene mutations in certain cancers, which may affect the structure and function of the MCM complex, thereby influencing the normal progression of DNA replication^27–31^. However, the majority of studies investigating the role of MCM4 in cancer have been confined to specific cancer types, thus creating a knowledge gap regarding its involvement across various cancers.

This research aims to comprehensively evaluate the expression patterns, functional implications, and diagnostic potential of MCM4 in various cancer types. To achieve this, we systematically analyzed data from the TCGA and GTEx datasets. Bioinformatic tools were then introduced to explore the expression profiles, genetic alterations, phosphorylation states, patterns of immune cell infiltration, immune subtypes, disease prognosis, as well as the diagnostic potential of MCM4 and its responsiveness to drugs in a range of cancers. In conclusion, this comprehensive study addresses a significant research gap by investigating the role of MCM4 in diverse cancer types and confirms that it may have translational implications for the diagnosis of cancer and strategies for its treatment.

## Results

### Structural characteristics and phylogenetic tree of MCM4

**Figure 1:**
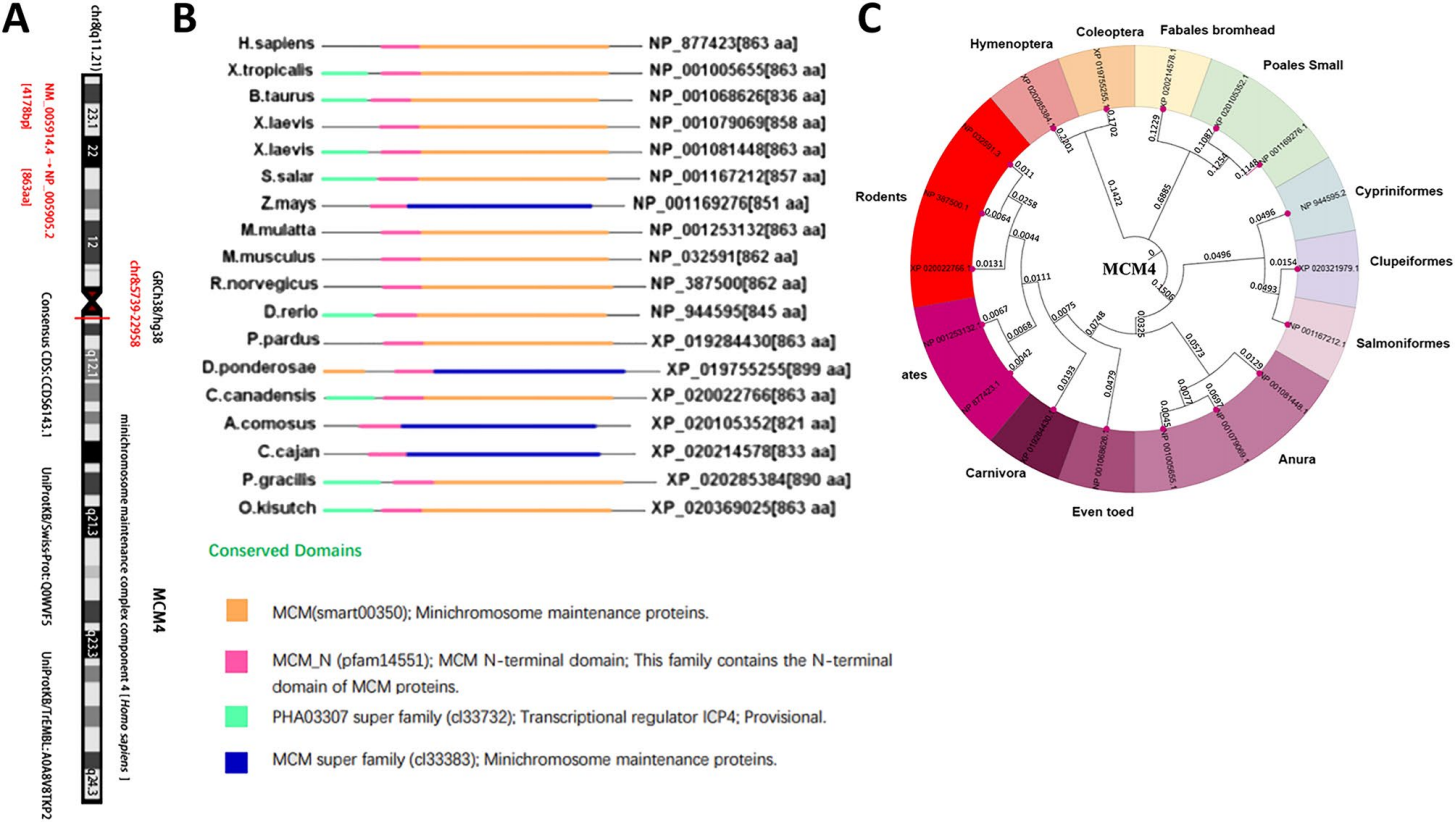
Structural characteristics and phylogenetic tree of MCM4. (**A**) The MCM4 gene is localized to the q11.21 region of chromosome 8; (**B**) The structural features of MCM4 proteins across different species; (**C**) Phylogenetic tree of MCM4.

In this study, our objective was to investigate the oncogenic role of human MCM4 (NM_005914.4 for mRNA and NP_005905.2 for protein, Fig. 1A). The structure of the MCM4 protein, as depicted in Fig. 1B, is highly conserved across different species and typically comprises the MCM (smart00350) domain and MCM N-terminal (pfam14551) domain. The phylogenetic tree depicts the evolutionary relationship of the MCM4 protein among various species (Fig. 1C).

### Differential expression of MCM4 in tumor and normal samples

**Figure 2:**
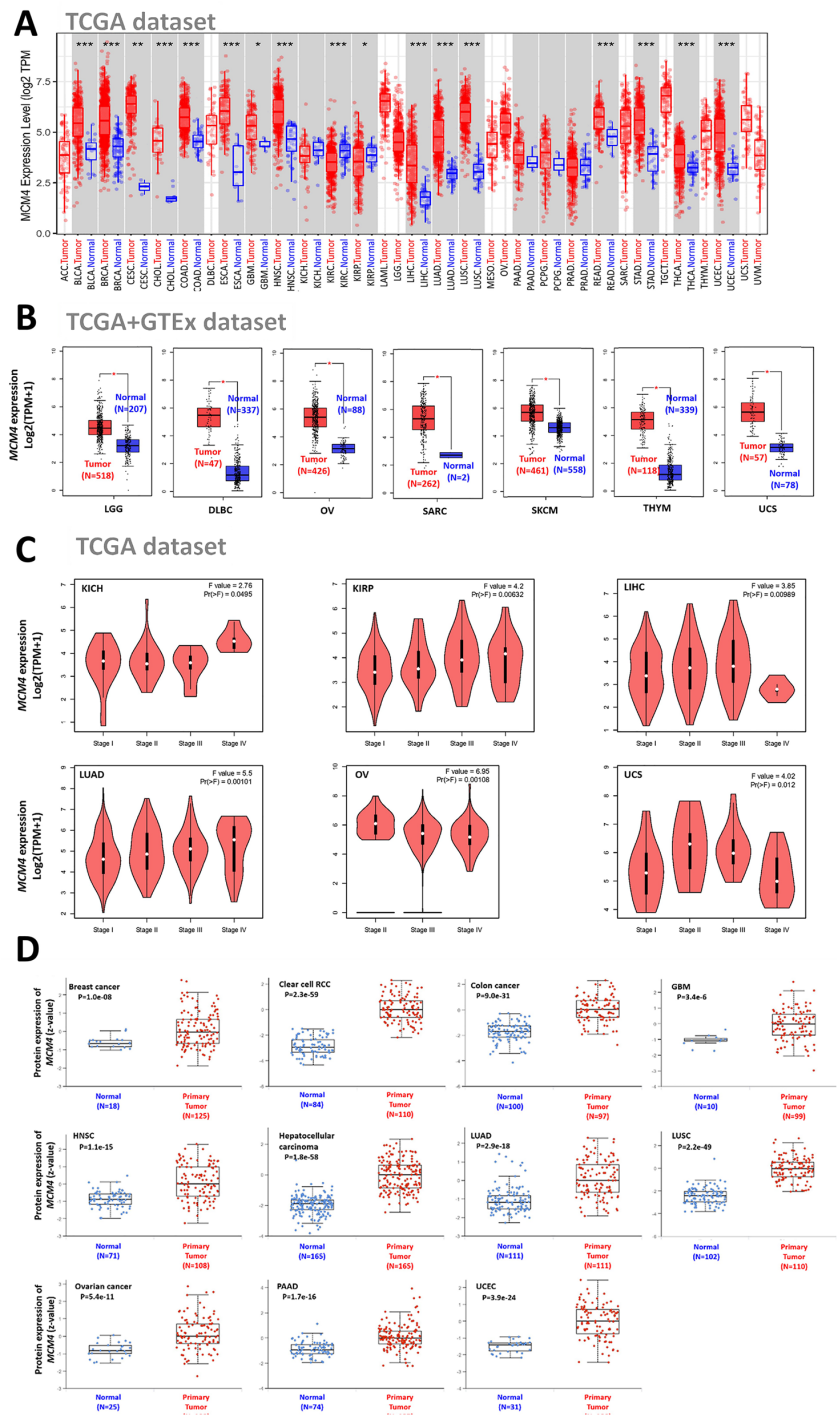
MCM4 expression across various cancers and pathological stages. (**A**) The transcription levels of MCM4 in various kinds of human cancers. Statistical significance was indicated as *P < .05, **P < .01, and ***P < .001. (**B**) In the TCGA project, for the types of LGG, DLBC, OV, SKCM, THYM, SARC and UCS, we used the corresponding normal tissues from the GTEx database as controls. (**C**) The expression levels of the MCM4 gene according to different pathological stages (stage I, II, III, and IV) in KICH, LUAD, OV, KIRP, LIHC and UCS. (**D**) The expression levels of total MCM4 protein between normal tissue and primary tumor tissue in KIRC, COAD, BRCA, GBM, LIHC, HNSC, LUSC, LUAD, OV, PAAD and UCEC.

In Fig. 2, we illustrate the expression levels of MCM4 in diverse human cancer types. We assessed the expression of MCM4 using the TIMER2 method on the TCGA dataset. Figure 2A presents an extensive depiction of the expression profiles of MCM4 in different cancers. Upregulation of MCM4 expression was observed in several cancer types, including BRCA, COAD, CESC, GBM, CHOL, HNSC, ESCA, LUAD, LIHC, READ, LUSC, STAD, LUSC, READ, THCA, and UCEC. Additionally, due to the lack of sufficient normal samples of OV, SARC, DLBC, LGG, SKCM, THYM and UCS in TCGA, normal tissues from the GTEx dataset were used as supplementary controls for this analysis, as shown in Fig. 2B (P < 0.05). Nevertheless, there were no notable variations observed in other cancer types, including ACC, KIRP, PRAD, LAML, KICH, KIRC, PCPG and TGCT (Fig. S1).

Furthermore, we employed the “Pathological Stage Plot” module of GEPIA2 to assess the association between MCM4 expression and cancer pathological stages across various cancer types. This analysis is depicted in Fig. 2C (all P < 0.05) and Fig. S2 (all displays no significance). Only in KICH, KIRP, LIHC, LUAD, OV, and UCS were there significant associations between MCM4 expression level and cancer stage.

To validate the expression levels of MCM4 at the protein level, we utilized the CPTAC dataset. Our analysis unveiled a significant upregulation of MCM4 protein expression in diverse cancer types when compared to normal tissues. These cancer types include BRCA, KIRC, HNSC, GBM, COAD, LUSC, LIHC, LUAD, OV, PAAD, and UCEC, as depicted in Fig. 2D. Unfortunately, other TCGA tumor types lack relevant protein data in the CPTAC database and this data gap needs to be filled. Furthermore, using IHC data obtained from the HPA database, we performed an analysis of MCM4 protein expression. Our findings, as depicted in Fig. S3, revealed significantly elevated levels of MCM4 protein expression in TGCT, CEST, COAD, BRCA, UCEC, LUAD, LIHC, STAD, BLCA, and SKCM.

**Figure 3:**
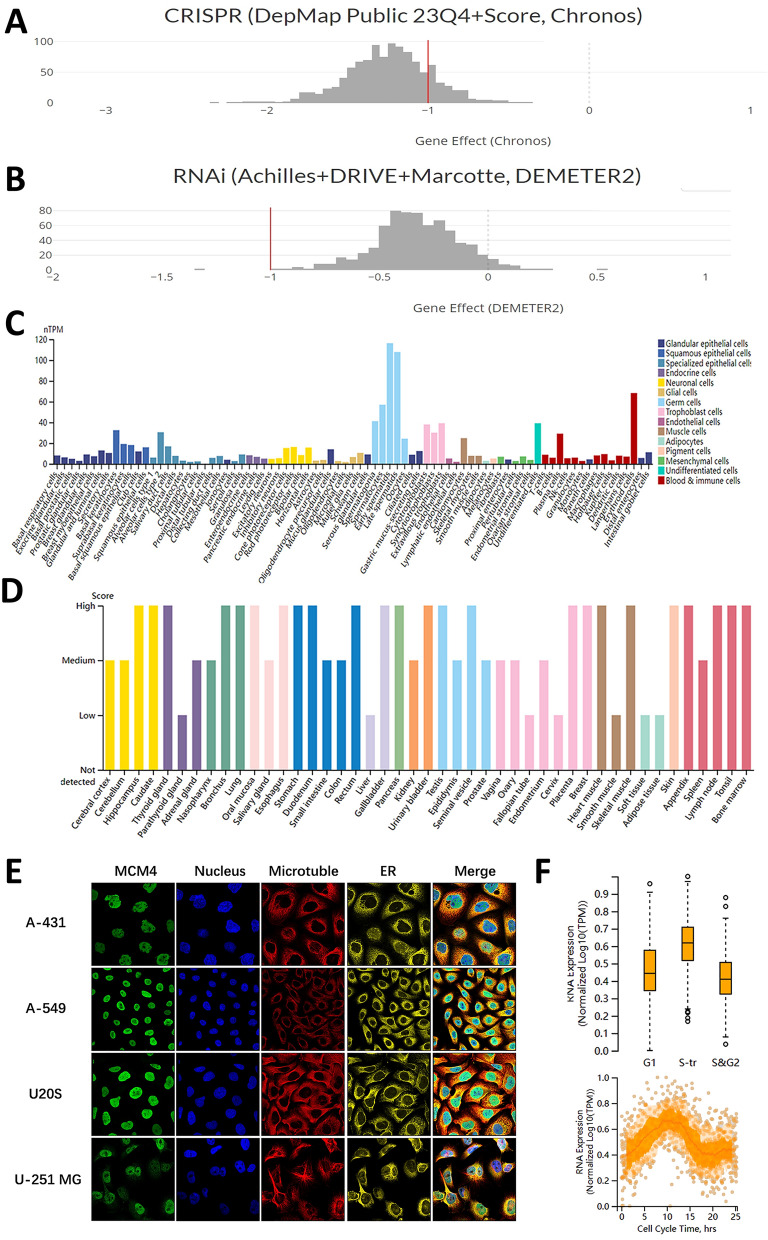
Single cell analysis of MCM4 in cancer and normal cell lines. (**A**) The overall essentiality of MCM4 in various cancer cell lines was demonstrated through CRISPR-Cas9 technology. (**B**) The overall essentiality of MCM4 in various cancer cell lines was demonstrated through RNAi assay. The Chronos and DEMETER2 dependency scores are derived from data obtained through a cellular depletion assay. In this context, a score of 0 indicates the non-essentiality of the gene, while a score of -1 corresponds to the median among all pan-essential genes, represented by the red line. (**C**) Normal cell line expression of MCM4. (D) MCM4 protein expression levels in various normal tissues. (**E**) Subcellular localization of MCM4 in the nucleus of A-431, A-549, U20S, and U-251MG cell lines. (**F**) The correlation between MCM4 transcript expression and progression of the cell cycle.

To substantiate the relevance of MCM4 across diverse cancer types, the validation process conducted via the DepMap database entailed an examination of its impact on multiple cancer cell lines at the cellular level. The employment of CRISPR for MCM4 knockdown and RNAi for interference with its expression elucidated a notable suppression of tumor cell growth in cancer cell lines across various cancer types (Fig. 3A,B). Specifically, the CRISPR study demonstrated a significant effect of MCM4 on tumor cells, evident in both the overall analysis and individual cell line assessments across all cancer types, with all gene effects registering below 0 and a mean effect skewed leftward of -1 (Fig. 3A, Fig. S4A). Correspondingly, the RNAi analysis corroborated these findings, albeit with marginally diminished significance compared to CRISPR. Both the overall effect and mean effect of tumor cell lines across diverse cancers remained sub-zero, although a few individual cell lines exhibited a non-essential effect (Fig. 3B, Fig. S4B). This discrepancy could potentially be attributed to CRISPR's ability to induce a more comprehensive loss of MCM4 function compared to RNAi.

Next, we proceeded to evaluate MCM4 expression in normal tissues. The expression pattern of MCM4 displayed significant variations across different cell types, with distinct disparities observed among various cellular compartments. Specifically, MCM4 exhibited notably lower expression levels in the great majority of cell lines, except in spermatogonia, spermatocytes, and erythroid cells, where its expression was comparatively elevated (Fig. 3C). Moreover, we observed moderate to high protein expression of MCM4 in diverse tissues, but its expression level was surprisingly low in parathyroid gland, liver, fallopian tube, cervix, smooth muscle, soft tissue, and adipose tissue (Fig. 3D). Furthermore, our investigation confirmed the subcellular localization of MCM4 within the endoplasmic reticulum (ER), microtubules, and nucleus of the A-431, A-549, U20S and U-251MG cell lines. Notably, MCM4 primarily localized in the nucleus, with no colocalization observed with the ER and microtubules (Fig. 3E). Additionally, by utilizing single-cell RNA sequencing data plotted using fluorescence ubiquitin-based cell cycle indicator (FUCCI), we identified a significant association between the advancement of G1, S, and G2 cell cycle stages and the expression level of MCM4 RNA (Fig. 3F).

### Gene mutation and protein phosphorylation of MCM4

**Figure 4:**
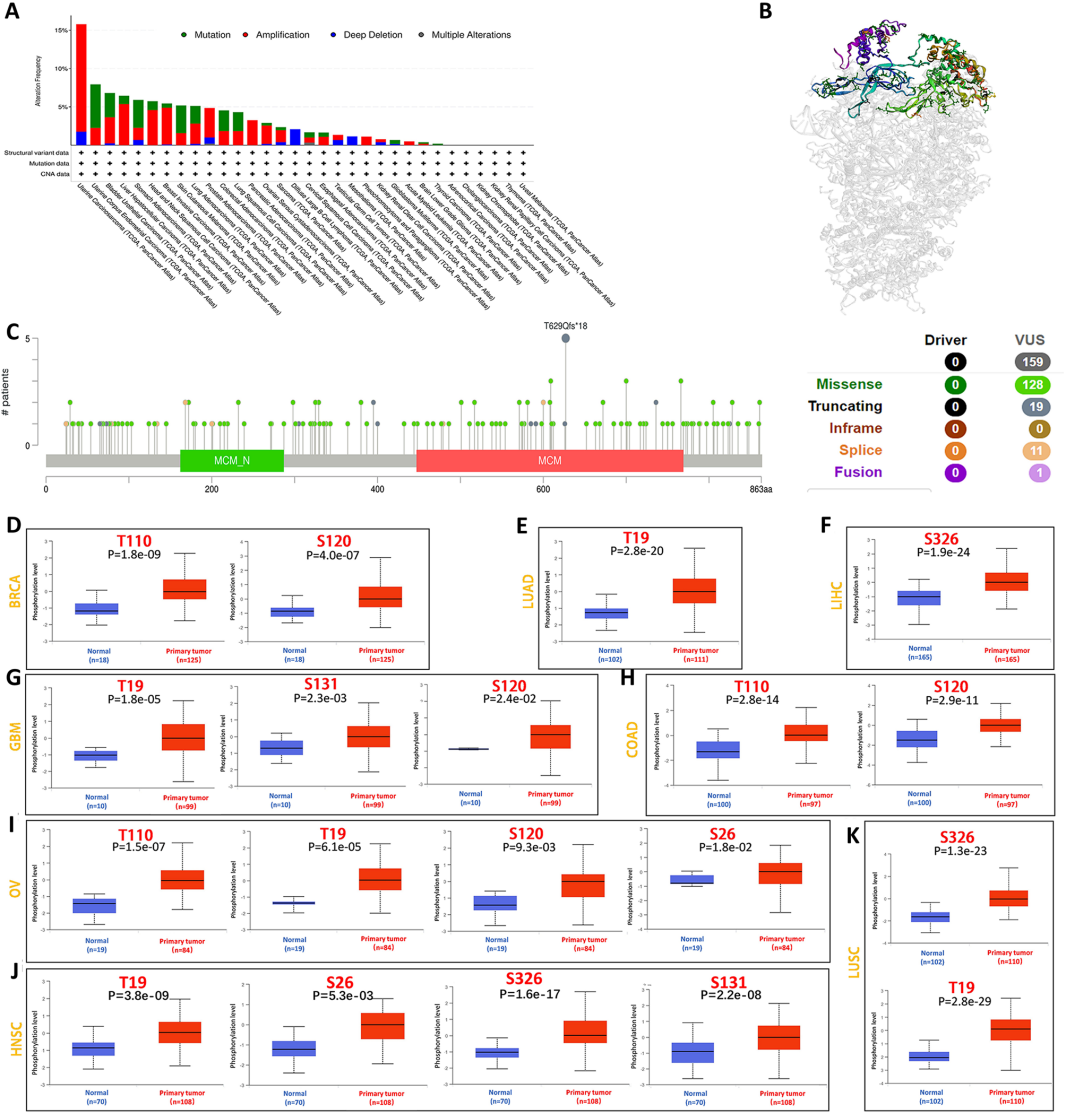
MCM4 gene mutation and post-translational phosphralation in diverse cancers. (**A**) Mutation rates and types of MCM4 in various cancers. (**B**) The three-dimensional structure refers to the spatial arrangement and conformation of the MCM protein. (**C**) Subtypes and distribution of somatic mutations in MCM4 gene. VUS, Variants of uncertain significance. (**D**) Phosphorylation site and level of MCM4 protein in BRCA, (**E**) LUAD, (**F**) LIHC, (**G**) GBM, (**H**) COAD, (**I**) OV, (**J**) HNSC and (**K**) LUSC.

Our aim was to investigate the frequency of somatic mutations in the MCM4 in various categories of cancer by examining a cohort of 32 tumor tissue samples. Research has found that MCM4 undergoes mutations in numerous cancer types, with the highest mutation rate observed in UCS, exceeding 15% (Fig. 4A). The majority of these mutations were amplifications or point mutations, leading to alterations in the primary protein structure or amino acid sequence. The three-dimensional structure of MCM4 is shown in Fig. 4B. Further analysis of the structure of MCM4 identified several mutation hotspots, with missense mutations being the predominant type (Fig. 4C). Specifically, the T629 residue was found to be the most frequently mutated site.

Afterwards, we assessed the correlation between MCM4 expression levels and various types of genomic alterations, including HRD, MATH, MSI, NEO, TMB, LOH, ploidy and purity, across all TCGA tumor samples. Our investigation revealed significant associations between MCM4 expression levels and various genetic alterations in specific cancer types. Specifically, we observed that high MCM4 expression levels correlated with increased HRD in GBM, LUAD, GBMLGG, LGG, KIPAN, KIRP, BRCA, SARC, PRAD, UCEC, LIHC, PAAD, HNSC, LUSC, BLCA, OV, ACC, and KICH (Fig. S5A). Additionally, we found a positive association. between MCM4 expression and MATH in LUAD, BRCA, ESCA, STES, HNSC, LUSC, MESO and BLCA, while GBMLGG, LGG, and KIRC showed a negative correlation (Fig. S5B). Furthermore, our results indicated a positive correlation between MCM4 expression and MSI in CESC, COADREAD, STES, COAD, GBM, SARC, KIPAN, STAD, KIRC, UVM, and CHOL, while GBMLGG, DLBC, THCA, and HNSC showed a negative correlation (Fig. S5C). We also detected a positive correlation between MCM4 expression and NEO in LUAD, COAD, COADREAD, and PRAD (Fig. S5D). In terms of tumor ploidy, we observed a positive correlation between MCM4 expression and ploidy in LUAD, BRCA, STES, SARC, STAD, PRAD, HNSC, LUSC, READ, SKCM, and BLCA. Conversely, GBMLGG, THCA, and OV exhibited a negative correlation (Fig. S5E). Interestingly, our research indicated a positive correlation between MCM4 expression and tumor purity in cancers such as GBM, LGG, CESC, BRCA, ESCA, STES, SARC, KIRP, STAD, UCEC, HNSC, LUSC, TGCT, PCPG, SKCM, and ACC, while THYM and READ showed a negative correlation (Fig. S5F). Additionally, we found a positive correlation between MCM4 expression and TMB in LUAD, COAD, COADREAD, STES, KIPAN, STAD, PRAD, and KICH (Fig. S5G). Finally, our study revealed a positive correlation between MCM4 expression and LOH in cancers such as GBM, SARC, KIRP, GBMLGG, LAML, BRCA, LGG, LUAD, ESCA, KIPAN, PRAD, LIHC, PAAD, UCEC, KIRC, LUSC, OV, UVM, BLCA, and CHOL, while THCA showed a negative correlation (Fig. S5H).

We further conducted a comparative analysis of the MCM4 phosphorylation levels in various primary tumor tissues and normal tissues utilizing the CPTAC dataset. Our findings indicate that the T19 locus demonstrates significantly elevated phosphorylation levels in five distinct primary tumor tissues, including GBM, HNSC, LUAD, LUSC, and OV. Additionally, the S120 gene locus displays increased phosphorylation levels in four primary tumor tissues. Notably, OV exhibits heightened phosphorylation levels at four gene loci: TP110, TP19, S120, and S26. Similarly, HNSC shows enhanced phosphorylation at four gene loci: TP19, S26, S326, and S131. Conversely, LUAD and LIHC display elevated phosphorylation levels at solely one gene locus, TP19 and S326, respectively (Fig. 4D–K).

### Molecular or immune subtypes of MCM4 in pan-cancer

**Figure 5:**
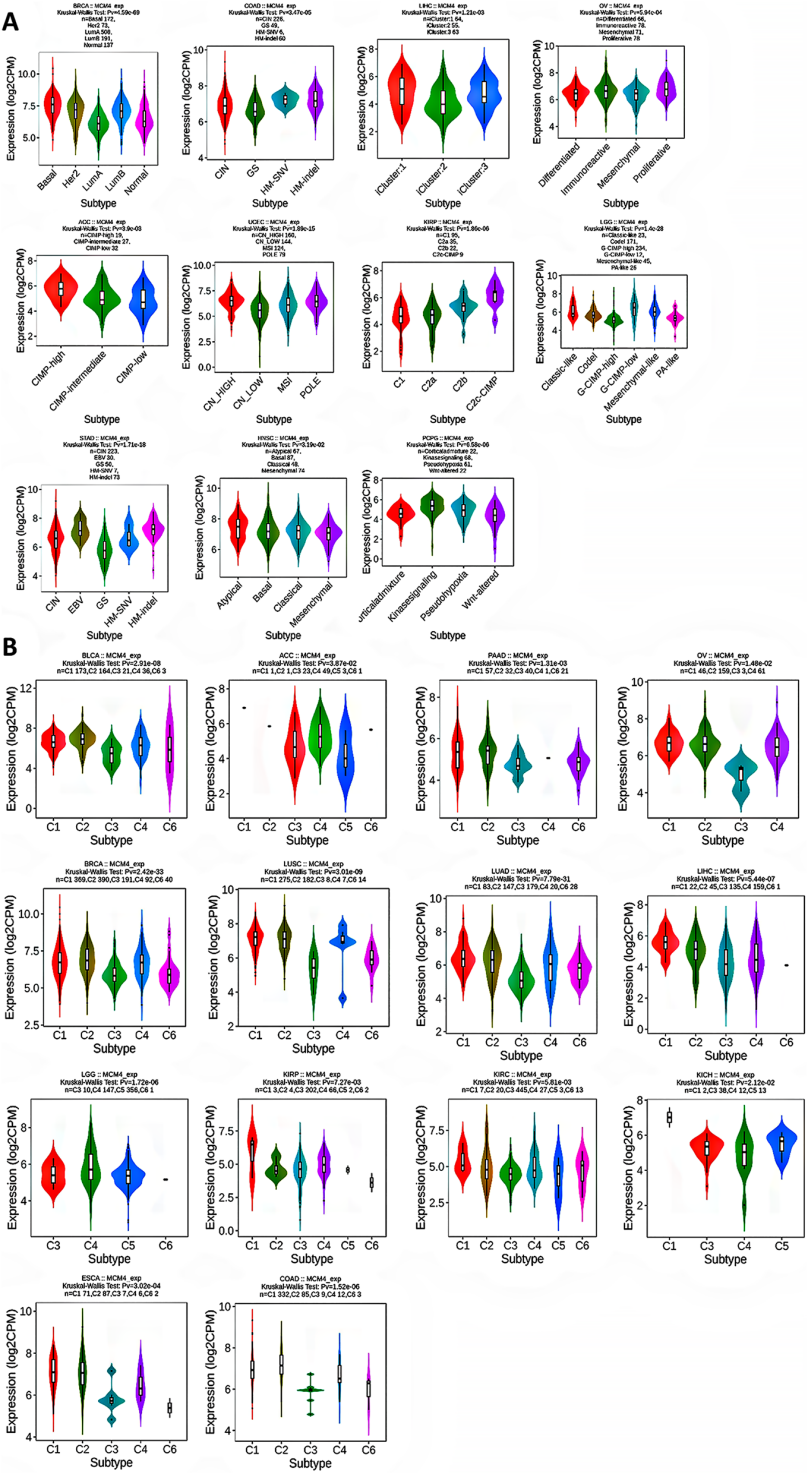
Associations between MCM4 expression and molecular subtypes and immune subtypes in TCGA cancers. (**A**) The relationship between MCM4 gene expression and various molecular subtypes in 11 types of TCGA cancers. (**B**) The association between MCM4 gene expression and immune subtypes in 14 types of TCGA cancers. *CIN* chromosomal instability, *GS* genomically stable, *POLE* Polymerase ε, *EBV* Epstein-Barr virus, *C1* wound healing, *C2IFN* gamma dominant, *C3* inflammatory, *C4* lymphocyte depleted, *C5* immunologically quiet, *C6* TGF-b dominant.

Eleven types of cancer were found to have molecular subtypes that are related to the expression of MCM4. Specifically, MCM4 expression was elevated in the basal molecular subtype of BRCA, while COAD displayed slightly increased MCM4 expression in both the HM-SNV and HM-indel subtypes. Notably, LIHC demonstrated heightened MCM4 expression in the iCluster:1 and iCluster:3 subtypes, and OV exhibited the highest levels of MCM4 expression particularly in the proliferative cell subtype. For ACC, the CIMP-high molecular subtype expresses MCM4 at the highest level. Additionally, UCEC showed marginally increased MCM4 expression in the CN_HIGH and POLE subtypes, while KIRP displayed peak MCM4 expression in the C2c-CIMP subtype. Furthermore, LGG presented higher MCM4 expression in the GCIMP-low subtype compared to other subtypes. For STAD, MCM4 was upregulated in the EBV and HM-indel subtypes. Last, HNSC revealed slightly elevated MCM4 expression in the atypical subtype, while PCPG showed maximum MCM4 expression in the kinase signaling subtype (Fig. 5A).

Immunotherapy, which harnesses the body's immune system to target and combat cancer cells, has proven efficacious in treating malignancies^32–34^. Accordingly, understanding the immune subtypes of various cancers is crucial for predicting treatment outcomes and optimizing therapeutic strategies. Remarkably, MCM4 expression was strongly correlated with immune subtypes across fourteen cancer types, including BLCA, ACC, PAAD, OV, BRCA, LUAD, KICH, LUSC, LGG, KIRP, LIHC, KIRC, ESCA and COAD (Fig. 5B).

### Correlations between MCM4 and immune infiltration in pan-cancer

**Figure 6:**
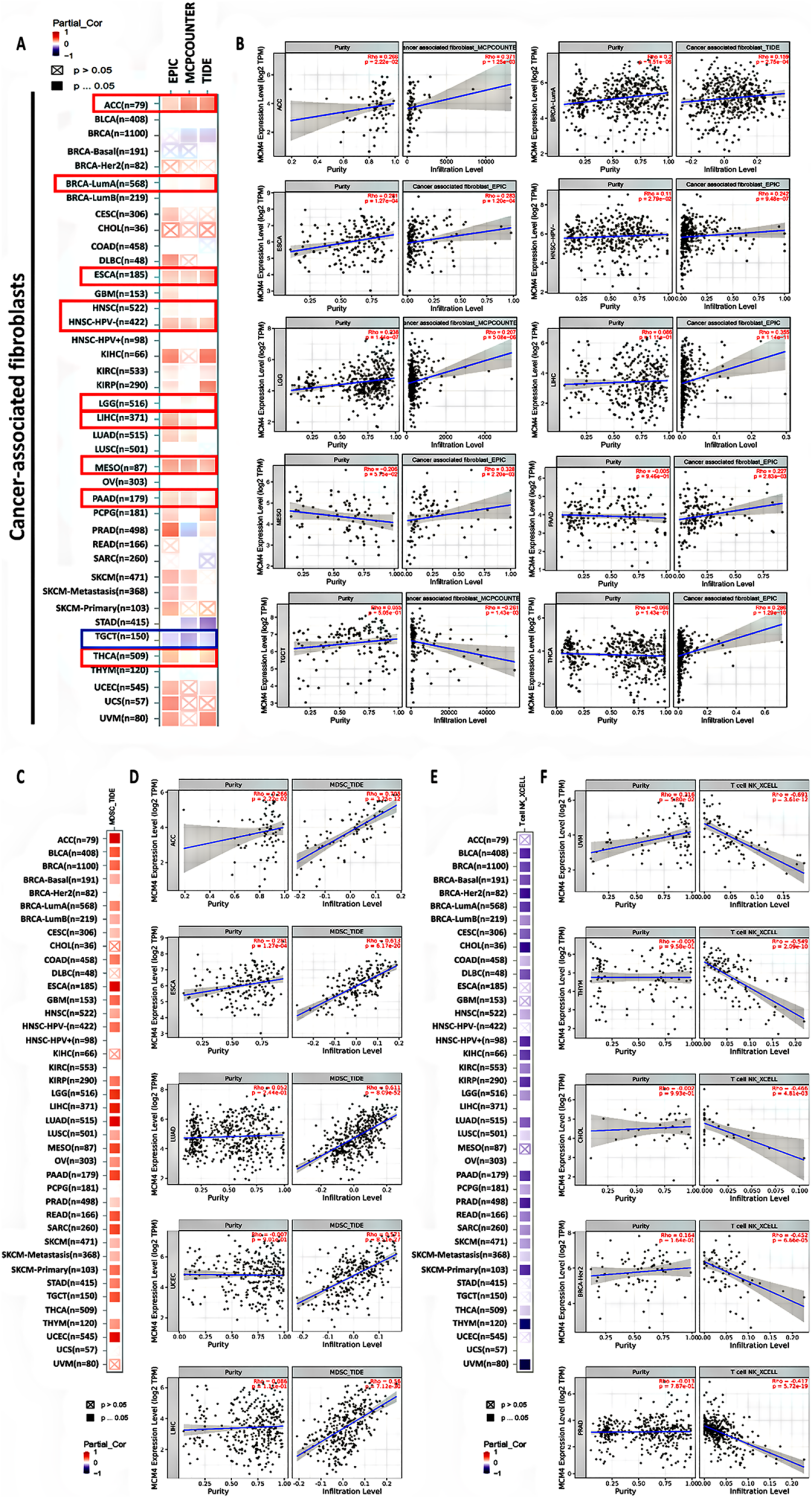
Correlation between MCM4 expression and immune cell infiltration in TCGA cancers. (**A**) Correlation between MCM4 expression and immune infiltration of cancer-associated fibroblasts, presented by heatmap. (**B**) Correlation between MCM4 expression and cancer-associated fibroblast infiltration in TGCT, BRCA-LumA, ACC, ESCA, HNSC(HPV-), LGG, MESO, PAAD, LIHC and THCA. (**C**) MCM4 expression exhibits a positive correlation with the infiltration of MDSCs and (**D**) top 5 tumors were ACC, ESCA, LUAD, UCEC and LIHC. (**E**) MCM4 expression shows a negative correlation with the infiltration of NKT cells and (**F**) top 5 tumors were UVM, THYM, CHOL, BRCA-Her2 and PRAD.

Immune cell infiltration of the tumor microenvironment can exert both positive and negative effects on cancer progression, depending on the functional status and type of infiltrating cells^35–37^. Consequently, we investigated the correlation between MCM4 expression and the level of immune infiltration in various immune cells across TCGA cancers. Our findings revealed a noteworthy and statistically significant positive association between the level of MCM4 expression and the estimated extent of cancer-associated fibroblast infiltration in TCGA tumors from several cancer types, including BRCA-LumA, ACC, ESCA, HNSC(HPV-), LGG, MESO, PAAD, LIHC and THCA. Conversely, a negative correlation was observed between MCM4 expression and cancer-associated fibroblast infiltration in TGCT (Fig. 6A,B). Moreover, our results revealed an intriguing pattern of correlations between the expression of MCM4 and the infiltration levels of two distinct immune cell populations in TCGA tumors. Specifically, a statistically significant positive correlation was noted between the level of MCM4 expression and the infiltration of myeloid-derived suppressor cells (MDSCs) in most of the TCGA tumors examined (Fig. 6C). The top 5 tumors were ACC, ESCA, LUAD, UCEC and LIHC (Fig. 6D). In contrast, a negative association was detected between MCM4 expression and the infiltration level of natural killer T cells (NKT) in most of the TCGA tumors (Fig. 6E) and the top 5 tumors were UVM, THYM, CHOL, BRCA-Her2 and PRAD (Fig. 6F). We conducted a more in-depth analysis on the correlation between MCM4 expression and the level of infiltration by six other immune cell types, specifically B cells, CD8 + T cells, CD4 + T cells, dendritic cells, macrophages, and neutrophils (Fig. S6A–F).

### Functional enrichment of MCM4 across cancers

**Figure 7:**
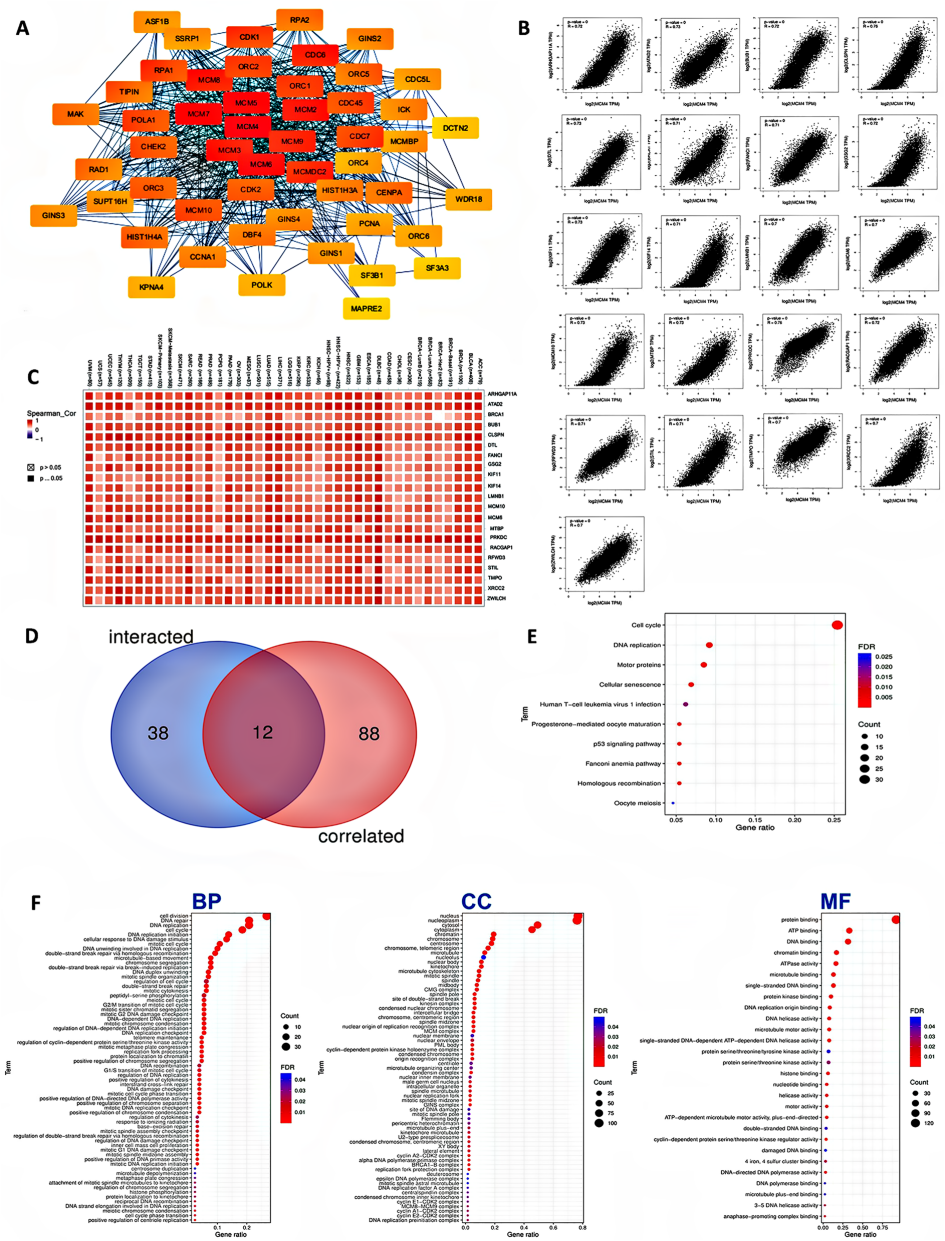
Functional and Pathway Enrichment Analysis of MCM4. (**A**) PPI network: 50 proteins interacting with MCM4 and their interaction networks. The darker the color of a node means that the node is more influential in the network. (**B**) Twenty-one genes with high correlation coefficients (R > 0.7) among a set of 100 genes known to be associated with MCM4 and (**C**) corresponding heatmap plot for various types of cancer. (**D**) Cross analysis between MCM4 binding genes and related genes, 12 identical genes are identified. (**E**) KEGG enrichment analysis. (**F**) GO enrichment analysis. *BP* Biological process, *CC* cellular component, *MF* molecular function.

To explore the molecular interactions and pathways associated with MCM4, we conducted a series of analyses to identify MCM4 binding proteins and genes related to MCM4 expression. Using the STRING, which relies on empirical evidence, we identified a total of 50 interacting proteins with MCM4. Then, using Cytoscape software, we mapped the PPI interactions network and explored the importance of a total of 51 nodes in the network (Fig. 7A). The darker color of a node means that the node is more influential in the network, and the influence ranking is also recorded simultaneously in the supplementary Table S1. The top 10 most important genes are MCM4, MCM7, MCM5, MCM6, MCM3, MCM2, MCM9, MCM8, MCMDC2, and ORC1, which are primarily members of the MCM gene family.

Subsequently, we utilized the GEPIA2 tool to integrate tumor expression data from TCGA and identified the top 100 genes that exhibited a correlation with MCM4 expression. Among these genes, 21 showed a strong positive correlation (R > 0.7), including ARHGAP11A, BUB1, DTL, GSG2, and others (Fig. 7B). This positive correlation was further supported by the heatmap data, which demonstrated its presence across various specific cancer types (Fig. 7C).

Subsequent cross-analysis of MCM4 correlative genes and interacting genes revealed 12 identical genes (Fig. 7D). We then performed KEGG and GO enrichment analyses to elucidate the underlying biological mechanisms. The KEGG analysis yielded 10 significantly enriched biological processes (p < 0.05), primarily revolving around cell cycle regulation, DNA replication, cellular sensitivity, and viral infection (Fig. 7E). In addition, GO enrichment analysis demonstrated that these genes are predominantly associated with DNA replication and cell division processes. Notably, cellular component analysis within GO enrichment identified diverse subcellular localizations for these genes, including nuclear and cytoplasmic compartments. Furthermore, molecular function analysis unveiled a significant enrichment of nucleotide, DNA, histone, and various enzyme binding implicated in DNA replication among MCM4 and related proteins (Fig. 7F).

To further examine the molecular mechanism of the MCM4 gene in tumor development, we employed a GGI network to uncover the correlation between MCM4 and its adjacent genes. From this analysis, we pinpointed 20 pertinent genes that displayed significant links with MCM4 (Fig. S7). These adjacent genes are mainly involved in regulating the initiation and extension of cell division, DNA replication, and cell cycle processes, as well as DNA repair, recombination, and telomere maintenance. They can also coordinate the response of cells to DNA damage.

### Clinical value of MCM4

**Figure 8:**
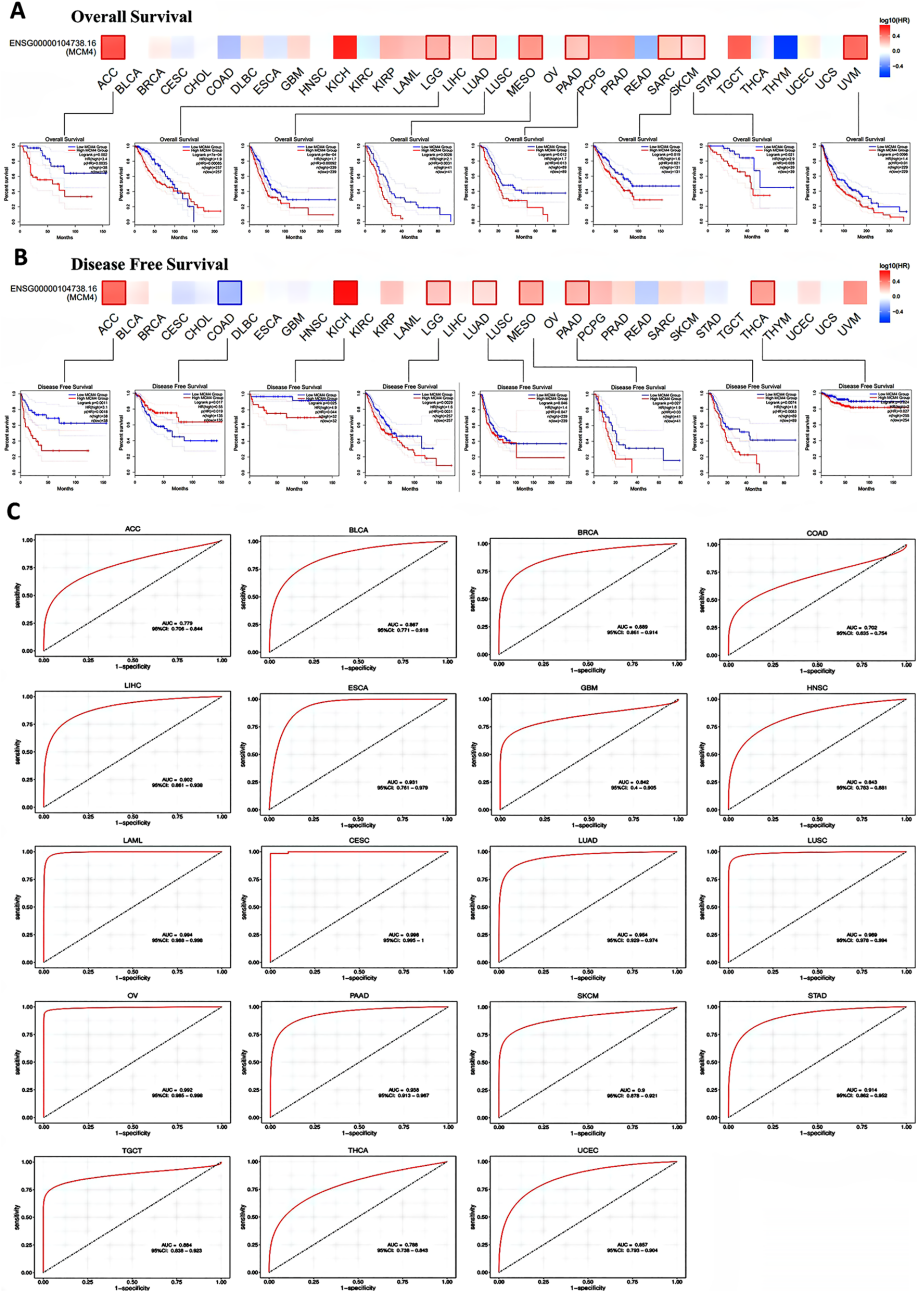
Clinical Value of MCM4. (**A**) Kaplan–Meier curves of overall survival rate and (**B**) disease-free survival rate exhibiting the association between MCM4 gene expression and cancer survival outcomes in TCGA. (**C**) The ROC curve of MCM4 for the diagnosis of different cancers. AUC of ROC curves verified the diagnosis performance of MCM4 in the TCGA cohort.

We aimed to evaluate the prognostic implications of MCM4 expression in various cancer types. Kaplan–Meier curves were generated to visualize the relationship between MCM4 expression and overall survival (OS) or disease-free survival (DFS) in each cancer type. As shown in Fig. 8A, increased MCM4 expression was significantly associated with unfavorable OS prognosis in several cancer types, including ACC (P = 0.0035), LGG (P = 0.00085), LUAD (P = 0.00092), MESO (P = 0.0031), PAAD (P = 0.013), SARK (P = 0.021), SKCM (P = 0.028), and UVM (P = 0.01). Similarly, high MCM4 expression was correlated with poor DFS prognosis in ACC (P = 0.0018), KICH (P = 0.044), LGG (P = 0.0031), LUAD (P = 0.047), MESO (P = 0.03), PAAD (P = 0.0083), and THCA (P = 0.027), as illustrated in Fig. 8B. Conversely, low expression of MCM4 was associated with unfavorable DFS prognosis in COAD (P = 0.019).

Utilizing ROC curves, we assessed the efficacy of MCM4 in forecasting the diagnosis of cancer patients. Our analysis encompassed 29 distinct cancers, with 4 cancers (CHOL, SARC, THYM, and UCS) demonstrating problematic results due to an insufficient number of normal samples. Additionally, 6 cancers (KICH, KIRC, KIRP, PCGC, PRAD, and READ) displayed AUC values below 0.7 (Fig. S8). The remaining 19 cancers were found to have AUC values above 0.7 when utilizing MCM4. MCM4 yielded exceptionally high predictive values in CESC (AUC = 0.998, 95% CI 0.995–1.000), LAML (AUC = 0.994, 95% CI 0.988–0.998), and OV (AUC = 0.992, 95% CI 0.985–0.998). Conversely, the predicted values of MCM4 were relatively low in the COAD prediction model (AUC = 0.702, 95% CI 0.635–0.754). Moreover, the predicted values of MCM4 spanned a range of 0.788 to 0.989 across the other 15 types of cancer (Fig. 8C).

Furthermore, we conducted a comprehensive analysis of the relationship between MCM4 expression and drug sensitivity across various cancer types using drug sensitivity genomics. Our findings revealed a positive correlation between MCM4 and 12 identified correlated interacting genes and five anticancer drugs, as well as a negative correlation with 25 drugs (Fig. S9). The magnitude of the correlation was found to be statistically significant, with larger dots representing stronger correlations. These results suggest that MCM4 may serve as a valuable biomarker for cross-cancer drug screening, enabling the identification of effective therapeutic strategies for treating diverse malignancies.

## Discussion

MCM4 functions as a constituent of the MCM2-7 complex, which plays a crucial role in DNA replication by serving as both a replication initiation factor and a potential replication helicase^38^. Research has provided evidence that the degradation of MCM4 can lead to a deficiency in MCM, causing disruptions in cellular proliferation and inducing genome instability^39^. Ultimately, these mechanisms contribute to the development of cancer and various developmental abnormalities^1,2^. Limited studies have employed bioinformatics approaches to examine the function of MCM4 in particular tumor types^21–25,40–42^, and a comprehensive pan-cancer analysis has yet to be undertaken. This investigation addresses this knowledge deficit by providing an exhaustive assessment of MCM4 across diverse cancer types, encompassing its expression levels in both normal and malignant tissues, gene mutation profiles, immune cell infiltration, involvement in biological processes, prognosis, potential as a diagnostic marker and drug sensitivity.

Our findings demonstrated a noteworthy increase in MCM4 expression across 22 various cancer types, in contrast to normal tissue. IHC analysis was also conducted to validate this conclusion. Our findings were further supported by the Depmap database's cancer dependency analysis of MCM4, which used RNA interference and CRISPR technology to show strong essentiality of MCM4 in a variety of cancer cell lines. We also detected a strong correlation between MCM4 expression and cancer progression. This association spanned the different pathological stages of six types of cancer, as well as the G1, S and G2 phases of the cell cycle. Our results, which are consistent with previous studies, suggest that MCM4 may contribute to the development of cancer^21,43–47^.

Existing studies have reported that exceptional phosphorylation and mutation of MCM4 have implications in the dysfunction of a variety of biological processes and may be contributors to cancer development^48–57^. Thus, we investigated the molecular structure and gene alterations of MCM4 to further explore the link between MCM4 and cancer. We found that mutations in MCM4 were frequent in cancer samples, and that phosphorylation levels of MCM4 were also elevated in 8 cancer types. In addition, we investigated 8 genetic characteristic indicators associated with tumors: HRD, MATH, MSI, NEO, ploidy, purity, TMB and LOH^58–66^. These indicators have been found to be strongly associated with the expression of MCM4. As these biomarkers can be used as targets for immunotherapy^67,68^, we speculate MCM4 is a promising drug target for anticancer immunotherapy.

From the perspective of precise medicine, immune and molecular subtypes of cancer are essential for diagnosis and personalized treatment^69–74^. In miscellaneous TCGA cancers, the correlation between MCM4 expression and molecular and immunological subtypes was found to be significant. The expression of MCM4 has been found to be associated with molecular subtypes in eleven different types of cancer and 14 types of immune classifications. These findings may underscore the potential significance of MCM4 expression in the diagnosis and treatment of cancer under the epoch of precision medicine.

Immune cell infiltration is of significant importance for understanding the immune evasion mechanisms of tumors and developing immunotherapy strategies. Extensive studies have indicated a close correlation between the degree and type of immune infiltration and the prognosis of tumors^35,75–78^. Previous research has revealed MCM4’s function in modulating immune cell activities especially for NKT cells^39,79,80^. We also investigated the association of MCM4 expression with immune infiltrates in different cancers. Cancer-associated fibroblasts have been proven to play several crucial roles in tumor development^81^. Our results indicated a positive correlation between MCM4 expression and the extent of infiltration of cancer-associated fibroblasts in various forms of cancer, suggesting that MCM4 may play a key role in promoting fibroblast recruitment into the tumor microenvironment. However, we also found that MCM4 expression was inversely associated with the degree of TGCT’s infiltration by cancer-associated fibroblasts. This finding suggests that MCM4 may have different functions in various types of cancer, and its role in TGCT may be distinct from its role in other cancer types. Understanding the molecular mechanisms underlying these differences requires further research. We also examined the correlation between MCM4 expression and the infiltration levels of ten distinct immune cell populations. Our results showed that MCM4 expression was positively correlated with the infiltration of MDSCs, but negatively correlated with the infiltration of NKT cells, while others showed no significant correlation. Overall, our study provides new insights into the relationship between MCM4 expression and immune infiltration in various cancer types. Future studies should continue to explore the functional implications of MCM4 expression in the context of cancer development and progression, with a focus on elucidating the molecular mechanisms underlying its effects on immune cell infiltration and tumor-immune cell interactions.

To elucidate the molecular mechanism underlying the role of MCM4 in tumor development, we constructed a PPI network and a GGI network around MCM4. We then performed an extensive analysis of KEGG and GO enrichment using the identified genes. Our findings provide insight into the potential role of MCM4 in modulating several cellular processes that contribute to cancer development and progression. First, our analysis revealed that MCM4 is involved in cell cycle regulation, cell proliferation, and DNA replication. These processes are tightly linked with cancer development, as uncontrolled cell growth and division can lead to the formation of tumors^82,83^. Second, our study found that MCM4 is part of a larger network of genes involved in various cellular processes, including cell signaling, metabolism, and apoptosis. This highlights the complexity of the molecular mechanisms of MCM4 underlying tumorigenesis^84,85^. In conclusion, our study provides evidence that MCM4 plays a crucial role in modulating various cellular processes that contribute to cancer development and progression. Future research should continue to explore the functional relationships between MCM4 and other genes in the context of cancer, with the goal of identifying novel mechanisms of tumorigenesis.

Finally, we explored the potential of MCM4 as a cancer biomarker and its correlation with poor prognosis in various cancers. Eight types of cancer were detected to have unfavorable OS prognoses while seven types of cancer had unfavorable DFS prognoses. We also evaluated the effectiveness of MCM4 in predicting the diagnosis of cancer patients. Strong diagnostic potential with AUC values above 0.7 was found in 19 types of cancer when utilizing MCM4 as a diagnostic biomarker. We finally analyzed the relationship between MCM4 expression and drug sensitivity in miscellaneous cancer types. Our findings indicate that MCM4 expression is positively correlated with resistance to five anticancer drugs including 17-AGG, PD-0325901, RDEA119, trametinib and selumetinib, and negatively correlated with sensitivity to 25 drugs. The significance of our study lies in the fact that MCM4 has not been previously studied extensively as a potential biomarker for cancer prognosis, diagnosis and drug sensitivity.

There are still several limitations to our study that should be acknowledged. First, due to the incomplete data in some databases, such as the lack of normal controls for certain cancer types in the TCGA data and the lack of corresponding data on protein expression levels and phosphorylation for certain TCGA cancer types in the CPTAC database, the comprehensiveness of our study will be affected. Second, our analysis was based on in silico data, which may not accurately reflect the clinical setting. Future studies should validate our findings using experimental models and clinical samples. Third, our study did not delve into the molecular mechanism underlying the role of MCM4 in cancers. Therefore, future research should be conducted to investigate the mechanism of MCM4 expression in tumors.

## Methods

### Molecular phylogenetic analysis

We searched for "MCM4" using the “Protein” Module on the NCBI website and selected protein sequences from species such as *H. sapiens*, *X. tropicalis*, *B. taurus* and others. We then analyzed the conserved domains of these proteins using the NCBI CD-Search tool and finally mapped these domains using the IBS website^86^. We obtained the amino acid sequence files of these proteins and drew the initial phylogenetic tree using MEGA11 software^87^.

### Expression analysis of MCM4

With the help of the TIMER2 database, we analyzed the differential mRNA expression levels of MCM4 across different cancers^88^. Wilcoxon’s test was employed to examine the statistical significance. In cases where normal tissue samples were limited or unavailable, we employed the "Expression analysis-box plot" module of the GEPIA2 database^89^. Our analysis was conducted with specific parameters, including a 0.05 P value cutoff, a log2FC (fold change) cutoff of 1, and the option to match TCGA and GTEx data.

To further explore the MCM4 level across different pathological stages, we utilized GEPIA2's “Pathological Stage Map” module. This module allowed us to generate violin plots based on log2 [TPM (Transcripts per Million) + 1] transformed expression data.

We also utilized the UALCAN portal, a dedicated web asset, to investigate the protein expression level by using the CPTAC module^90,91^. Our focus was on examining the expression status of the MCM4 protein in both primary tumor tissues and normal tissues separately. Additionally, immunohistochemistry (IHC) data were retrieved from the Human Protein Atlas (HPA) database^92^.

We performed an exhaustive assessment of the protein levels and transcript abundance of MCM4 in various normal tissues and cell lines, utilizing the extensive resources available through the HPA. Furthermore, we carried out a analysis of the subcellular localization of MCM4 by obtaining immunofluorescence (IF) data from the HPA database. Moreover, to gain additional insights into the involvement of MCM4 in cellular mechanisms, we examined the association between the expression of MCM4 transcripts and the progression through the cell cycle.

### Cancer dependency analysis

DepMap (Cancer Dependency Map) is a database used to screen potential therapeutic targets for cancer by utilizing RNAi and CRISPR-Cas9 technology^93,94^. Through DepMap database, we obtained the gene dependency data of MCM4 in various cancer cell lines. The Chronos and DEMETER2 dependency scores rely on data derived from a cellular depletion assay. A reduced Chronos or DEMETER2 score suggests an increased probability that the gene under examination is crucial within a specific cell line. A score of 0 denotes non-essentiality of the gene, while -1 aligns with the median among all pan-essential genes (represented by the red line).

### Genetic alternation analysis

Using the cancer genomics database cBioPortal, we conducted a comprehensive exploration of the specific mutation types and sites involved in MCM4 amino acids across various types of cancer^95^. Three-dimensional (3D) model was also obtained from cBioportal.

To further investigate the correlation between MCM4 expression and diverse tumor genomic characteristics including tumor mutational burden (TMB), microsatellite instability (MSI), homologous recombination deficiency (HRD), mutant allele tumor heterogeneity (MATH), neoantigen, purity, ploidy and loss of heterozygosity (LOH), the sangerBox website was utilized.

### Protein phosphorylation analysis

We utilized the online platform UALCAN to perform a detailed analysis of phosphoprotein expression^90,91^. Our investigation focused on quantifying the levels of both total protein and phosphoprotein expression of MCM4 across various cancer types.

### Molecular or immune subtypes of MCM4

TISIDB database, a comprehensive resource that integrates various data formats to assess interactions between tumors and the immune system^96^. Our analysis concentrate on investigating the connections between MCM4 expression levels and molecular or immune subtypes in multiple cancers.

### Immune infiltration analysis

For investigation of the correlation between MCM4 expression and immune infiltration in various TCGA tumor types, we utilized the “Immune Genes” module of the TIMER2 webserver. We evaluated the strength of association by utilizing the purity-corrected Spearman's rank correlation test and provided Partial_cor values and P-values. A scatter plot and heatmap were used to present the results.

### PPI network and GGI network analysis

In order to predict the functional association between MCM4 and genes with similar functions, we utilized the GeneMANIA website to construct a gene–gene interaction (GGI) network^97^. Additionally, we used STRING website to identify the binding protein associated with MCM4. This led to the formation of a protein–protein interaction (PPI) network^98^. To ensure the reliability of the network, we applied several filtering criteria, including a minimal required interaction score of 0.150, consideration of significant network boundaries with evidence support, limitation of displayed interactors was limited to 50 interactors, and utilization of active experimental sources as interaction inputs. To further indentify the most influential node in the network, genes were analyzed using and ''CytoHubba'' app in Cytoscape (edition 3.10.1). All 51 genes in the network was ranked by “Degree” method.

### Functional enrichment analysis

To identify genes that are closely correlated with MCM4, we exploited the "Similar Gene Detection" module of GEPIA2. By inputting MCM4 as the gene of interest, we obtained a list of the top 100 genes that exhibited strong correlation with MCM4 expression. In addition, we performed pairwise Pearson correlation analysis on the top 100 associated genes of MCM4, which led to the identification of 21 genes exhibiting a strong positive correlation (R > 0.7). Point plots were generated to visualize the correlation coefficients (R) and corresponding P values. Subsequently, we exploited the "GeneCorr" module available on the TIMER2 platform to generate heatmap that included partial correlation coefficients (cor) and corresponding P values obtained from the Spearman rank correlation test. Finally, we leveraged a Venn plot for cross-analysis to identify overlapping genes among the set of interacting and relating genes.

We couducted functional enrichment analysis utilizing the Kyoto Encyclopedia of Genes and Genomes (KEGG) and Gene Ontology (GO) databases^99,100^. We employed the R programming language and the ggplot2 and cluster packages to execute this analysis. Specifically, we utilized the cnetplot function to visualize the data from the cellular component (CC), biological process (BP) and molecular function (MF) categories of GO, with circular layout disabled (circular = F) and edge colors enabled (colorEdge = T). Additionally, we opted for node labels to be displayed (node_label = T) for improved interpretability.

### Survival analysis

We utilized the "survival plot" module supplied by GEPIA2^89^. The expression data were clustered into high and low groups using a threshold-based method, where the threshold value was set at 50%. With the assumption that the log-rank test was employed for survival analysis, we generated survival plots.

### Receiver operating characteristic analysis

We initially obtained expression data from both normal and cancerous tissues from the TCGA and GTEx databases and then conducted receiver operating characteristic (ROC) assays using the “R pROC” and “R ggplot2” packages in R studio. This analysis involved calculating the area under the curve (AUC) scores.

### Drug sensitivity analysis

GSCALite incorporates information on over 750 small-molecule medications from the GDSC and CTRP databases^101^. To evaluate the sensitivity of the MCM4 gene and its interactions with other genes in relation to multiple anticancer drugs, GSCALite was used.

### Supplementary Information

Supplementary Information.

## Data Availability

In this study, publicly available datasets were analyzed. All data can be found: The UCSC XENA database (https://xenabrowser.net/datapages/), the TCGA Pan-Cancer datasets (https://xenabrowser.net/), the Genotype-Tissue Expression database (https://www.genome.gov/Funded-Programs-Projects/Genotype-Tissue-Expression-Project), the NCBI website (https://www.ncbi.nlm.nih.gov/), the MEGA11 software (https://megasoftware.net/), the Human Protein Atlas database (https://www.proteinatlas.org/), the UALCAN portal (http://ualcan.path.uab.edu/analysis-prot.html), the GEPIA2 database (http://gepia2.cancer-pku.cn/#index), the cBioPortal (https://www.cbioportal.org/), the Tumor Immune Estimation Resource version 2 (TIMER2) (http://timer.cistrome.org/), the STRING tool (https://string-db.org/) and the DepMap (Cancer Dependency Map) Portal (https://depmap.org/portal/).

## Abbreviations

ACC: Adrenocortical carcinoma
BLCA: Bladder urothelial carcinoma
BRCA: Breast invasive carcinoma
CESC: Cervical squamous cell carcinoma and endocervical adenocarcinoma
CHOL: Cholangiocarcinoma
COAD: Colon adenocarcinoma
DLBC: Lymphoid neoplasm diffuse large B-cell lymphoma
ESCA: Esophageal carcinoma
GBM: Glioblastoma multiforme
HNSC: Head and neck squamous cell carcinoma
KICH: Kidney chromophobe
KIRC: Kidney renal clear cell carcinoma
KIRP: Kidney renal papillary cell carcinoma
LAML: Acute myeloid leukemia
LGG: Brain lower grade glioma
LIHC: Liver hepatocellular carcinoma
LUAD: Lung adenocarcinoma
LUSC: Lung squamous cell carcinoma
MESO: Mesothelioma
OV: Ovarian serous cystadenocarcinoma
PAAD: Pancreatic adenocarcinoma
PCPG: Pheochromocytoma and paraganglioma
PRAD: Prostate adenocarcinoma
READ: Rectum adenocarcinoma
SARC: Sarcoma
SKCM: Skin cutaneous melanoma
STAD: Stomach adenocarcinoma
TGCT: Testicular germ cell tumors
THCA: Thyroid carcinoma
THYM: Thymoma
UCEC: Uterine corpus endometrial carcinoma
UCS: Uterine carcinosarcoma
UVM: Uveal melanoma
