# Supplementary material for: Multi-omics pan-cancer analyses identify MCM4 as a promising prognostic and diagnostic biomarker
Source: Sci Rep. 2024 Mar 18;14:6517. doi: 10.1038/s41598-024-57299-1 (PMC10948783; doi:10.1038/s41598-024-57299-1)
Supplement: Supplementary file 1 — Supplementary Information. [file 41598_2024_57299_MOESM1_ESM.pdf]

Supplementary Material

Supplementary Figures

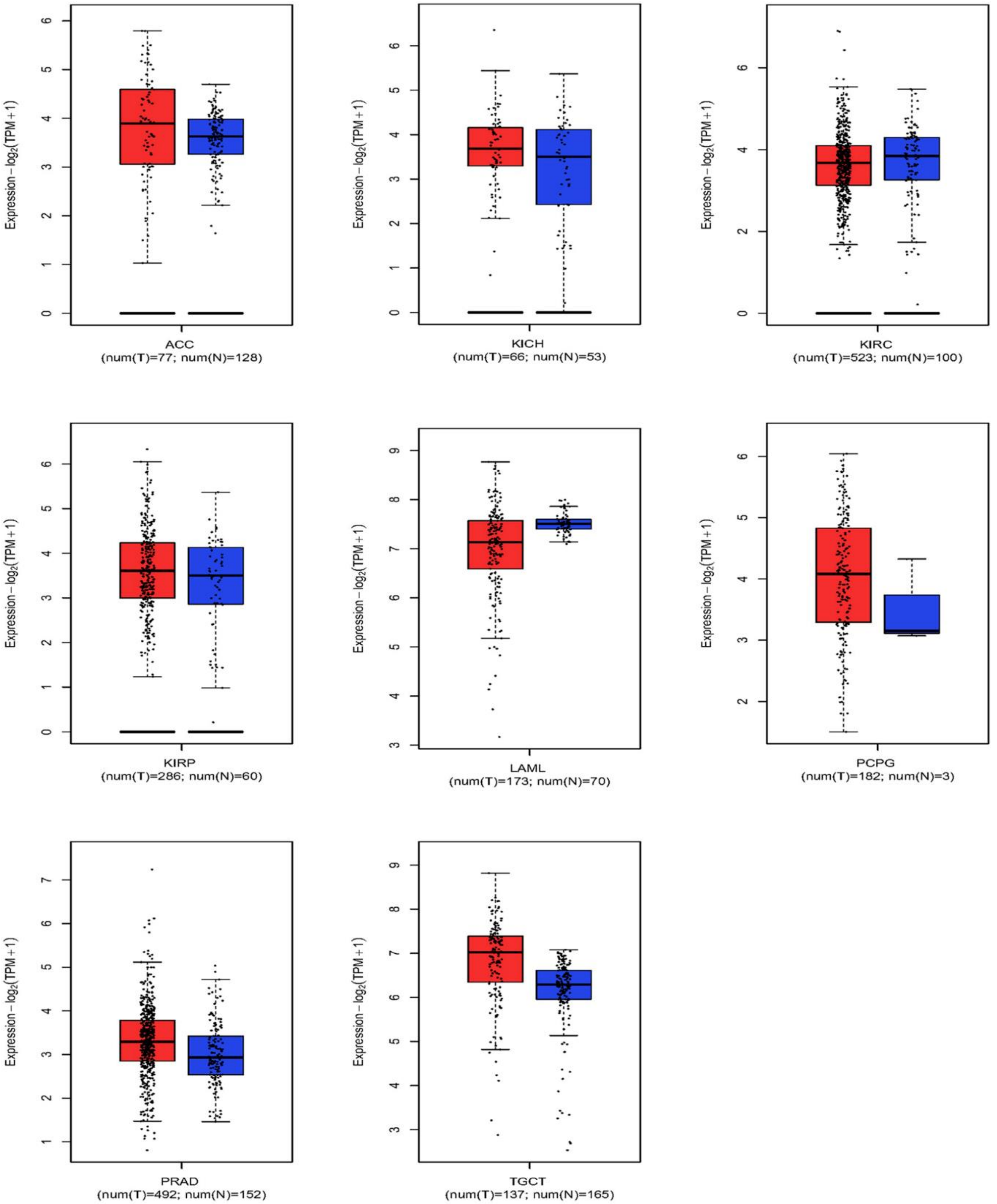

Figure S1. The expression levels of the MCM4 in ACC, KICH, KIRC, KIRP, LAML, PCPG, PRAD and TGCT. Data derived from TCGA and GTEx databases.

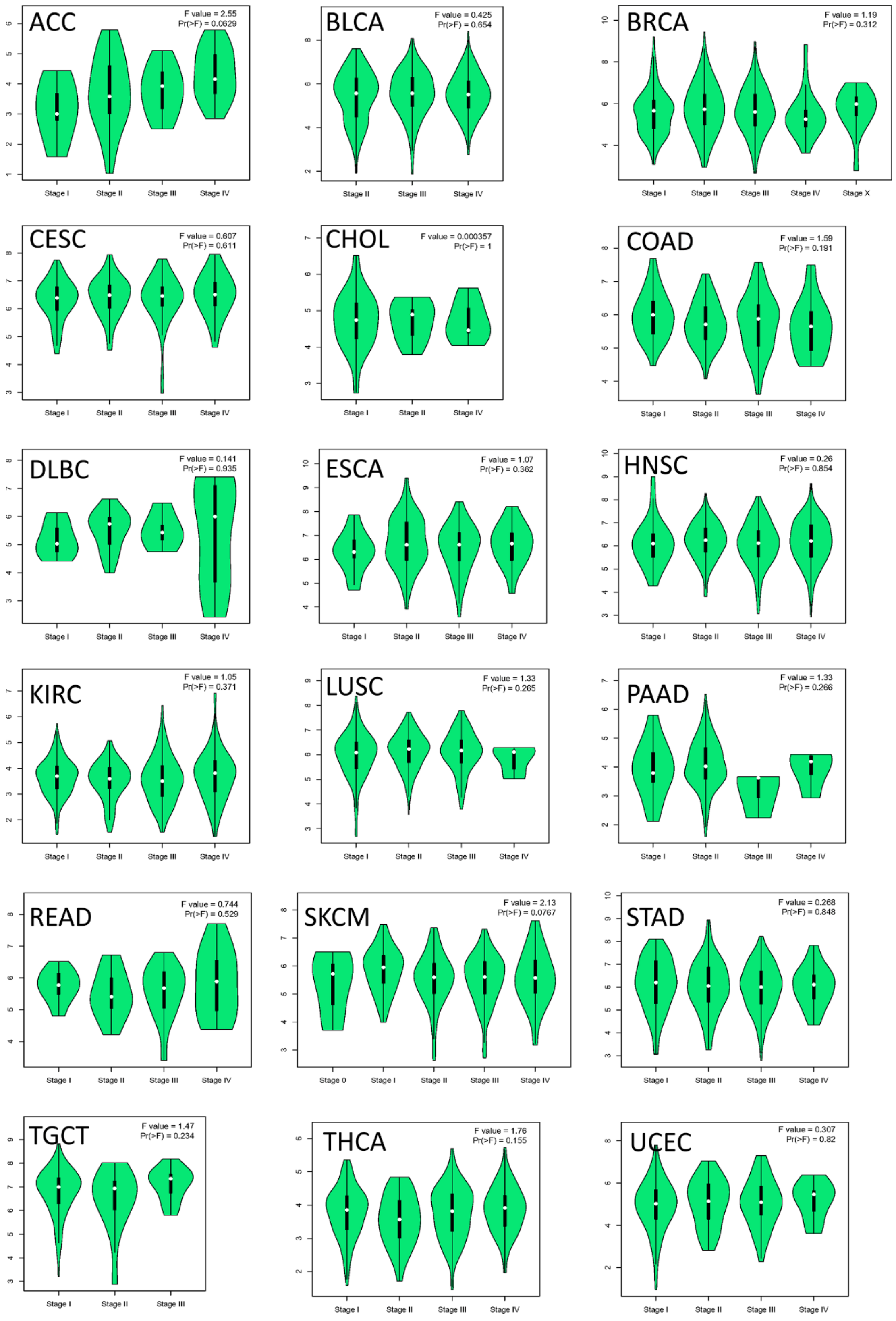

Figure S2. Expression levels of the MCM4 in different pathological stages of ACC, BLCA, BRCA, CESC, CHOL, COAD, DLBC, ESCA, HNSC, KIRC, LUSC, PAAD, READ, SKCM, STAD, TGCT, THCA and UCEC.

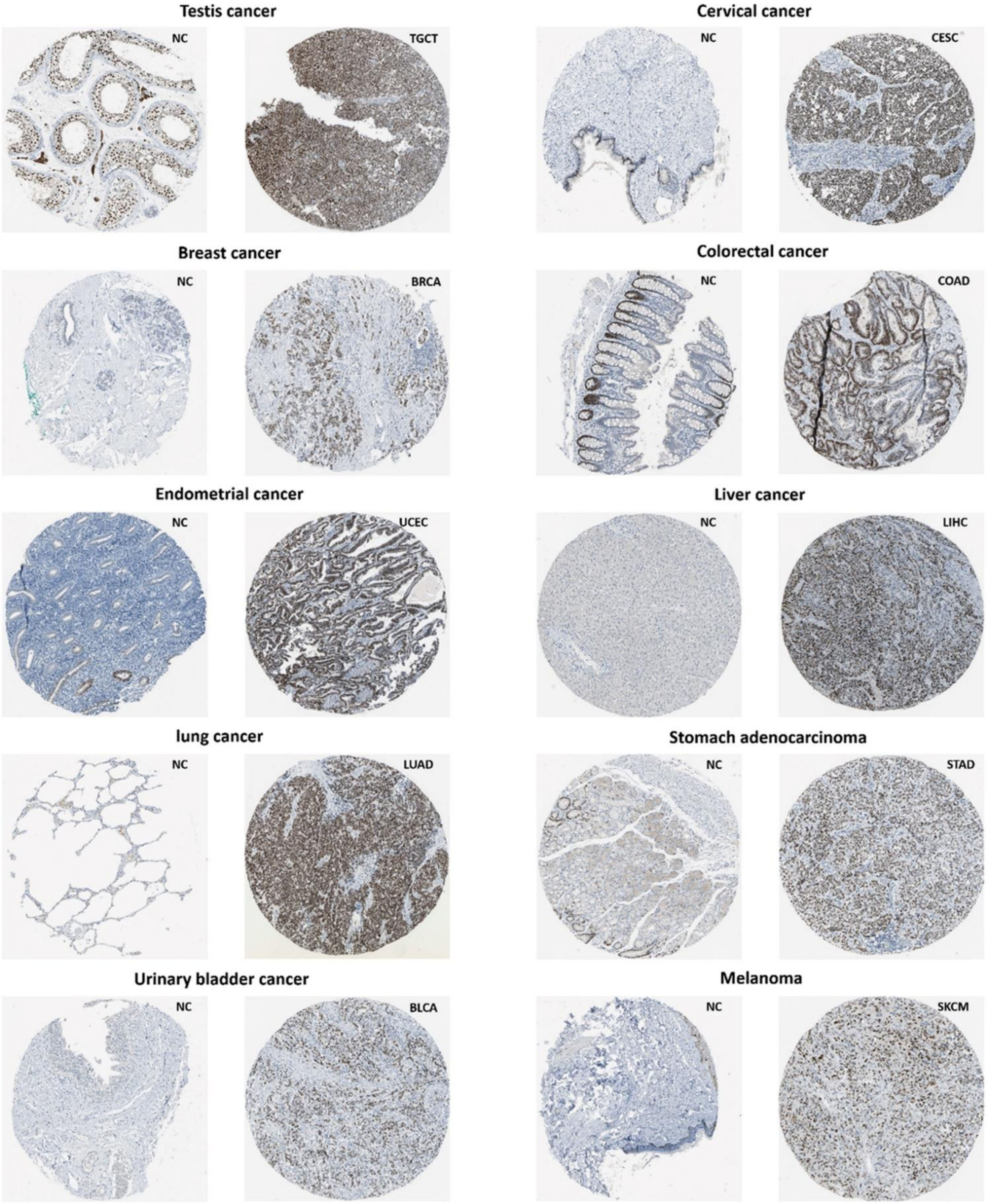

Figure S3. Representative immunohistochemistry images were used to contrast the expression of MCM4 between normal tissue and tumor tissue in TGCT, CESC, BRCA, COAD, UCEC, LIHC, LUAD, STAD, BLCA, and SKCM.

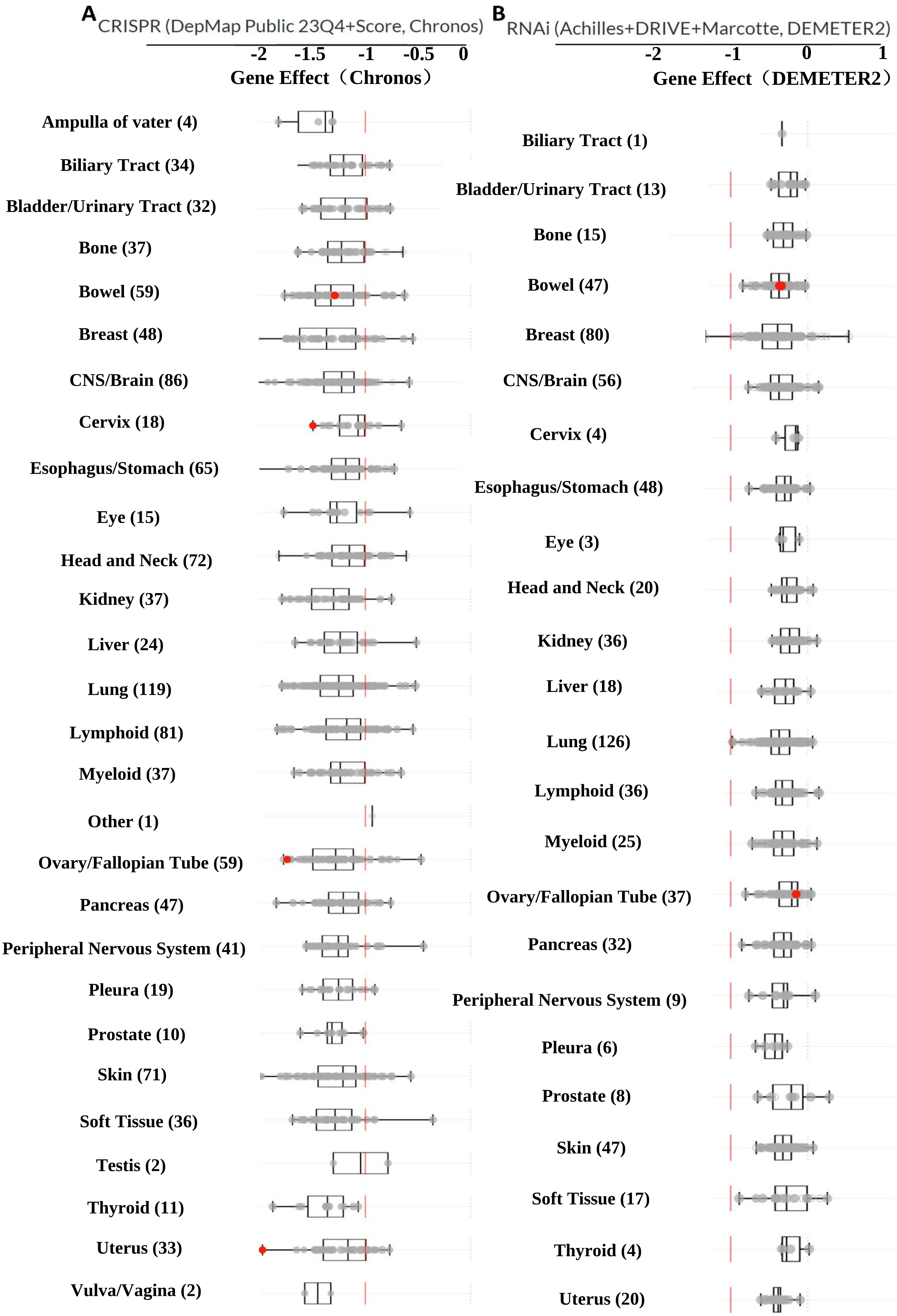
A
CRISPR (DepMap Public 23Q4+Score, Chronos)
-2 -1.5 -1 -0.5 0
Gene Effect（Chronos）
Ampulla of vater (4)
Biliary Tract (34)
Bladder/Urinary Tract (32)
Bone (37)
Bowel (59)
Breast (48)
CNS/Brain (86)
Cervix (18)
Esophagus/Stomach (65)
Eye (15)
Head and Neck (72)
Kidney (37)
Liver (24)
Lung (119)
Lymphoid (81)
Myeloid (37)
Other (1)
Ovary/Fallopian Tube (59)
Pancreas (47)
Peripheral Nervous System (41)
Pleura (19)
Prostate (10)
Skin (71)
Soft Tissue (36)
Testis (2)
Thyroid (11)
Uterus (33)
Vulva/Vagina (2)
B
RNAi (Achilles+DRIVE+Marcotte, DEMETER2)
-2 -1 0 1
Gene Effect（DEMETER2）
Biliary Tract (1)
Bladder/Urinary Tract (13)
Bone (15)
Bowel (47)
Breast (80)
CNS/Brain (56)
Cervix (4)
Esophagus/Stomach (48)
Eye (3)
Head and Neck (20)
Kidney (36)
Liver (18)
Lung (126)
Lymphoid (36)
Myeloid (25)
Ovary/Fallopian Tube (37)
Pancreas (32)
Peripheral Nervous System (9)
Pleura (6)
Prostate (8)
Skin (47)
Soft Tissue (17)
Thyroid (4)
Uterus (20)

Figure S4. Essentiality of MCM4 in cancer cell lines. **(A)** MCM4's essentiality in cancer cell lines across various cancer types, verified by CRISPR knockdown. **(B)** MCM4's essentiality in cancer cell lines across various cancer types, verified by RNAi interference. The number in parentheses in each row corresponds to the number of tumor cell line types belonging to that tissue or organ recorded in the database. Each solid gray dot corresponds to a cancer cell line.

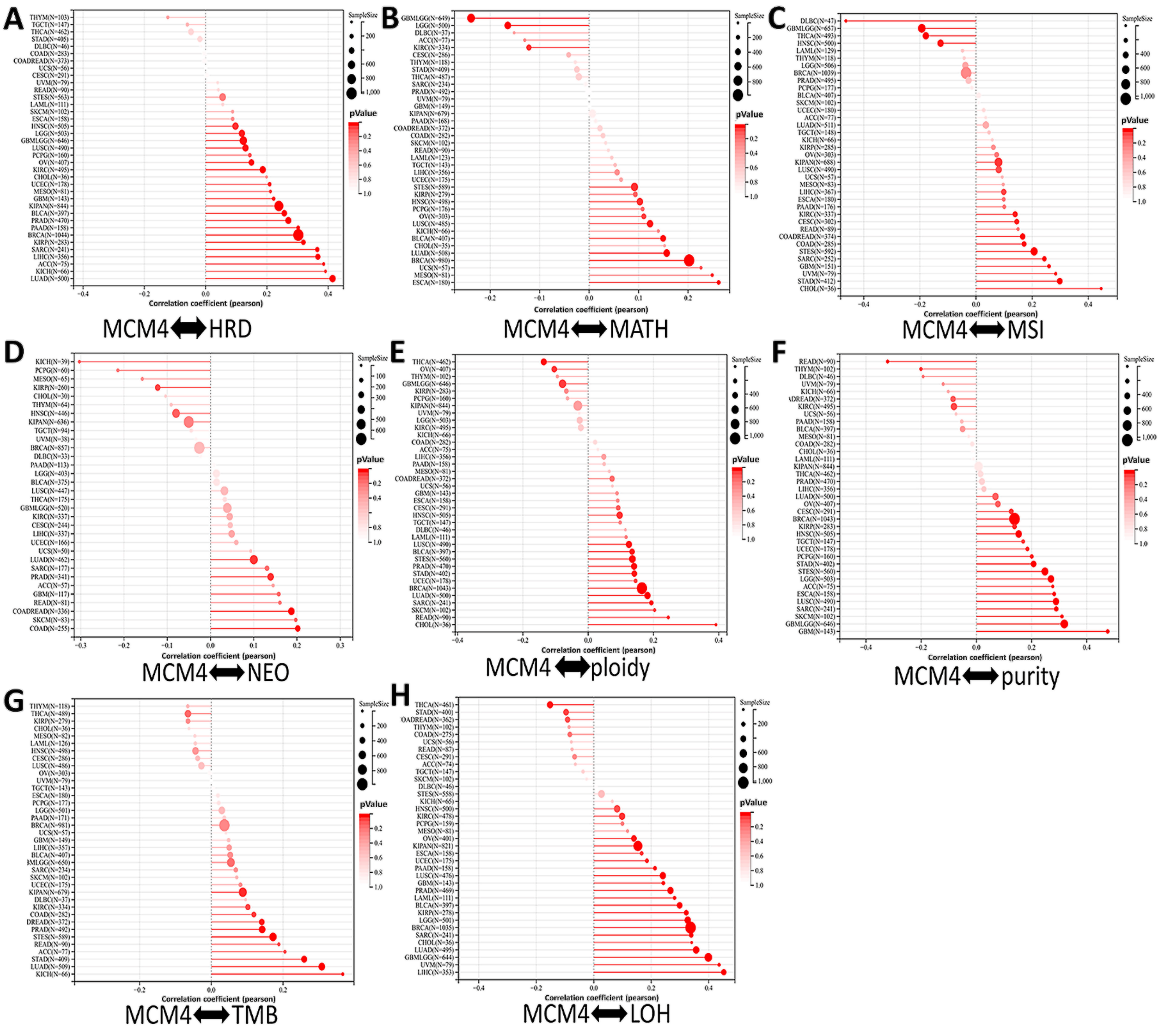

Figure S5. Correlation between MCM4 gene expression level and genetic characteristic indicators associated with tumors, including (**A)** HRD, **(B)** MATH, **(C)** MSI, **(D)** NEO, **(E)** ploidy, **(F)** purity, (G) TMB and (H) LOH, across all TCGA tumor samples.

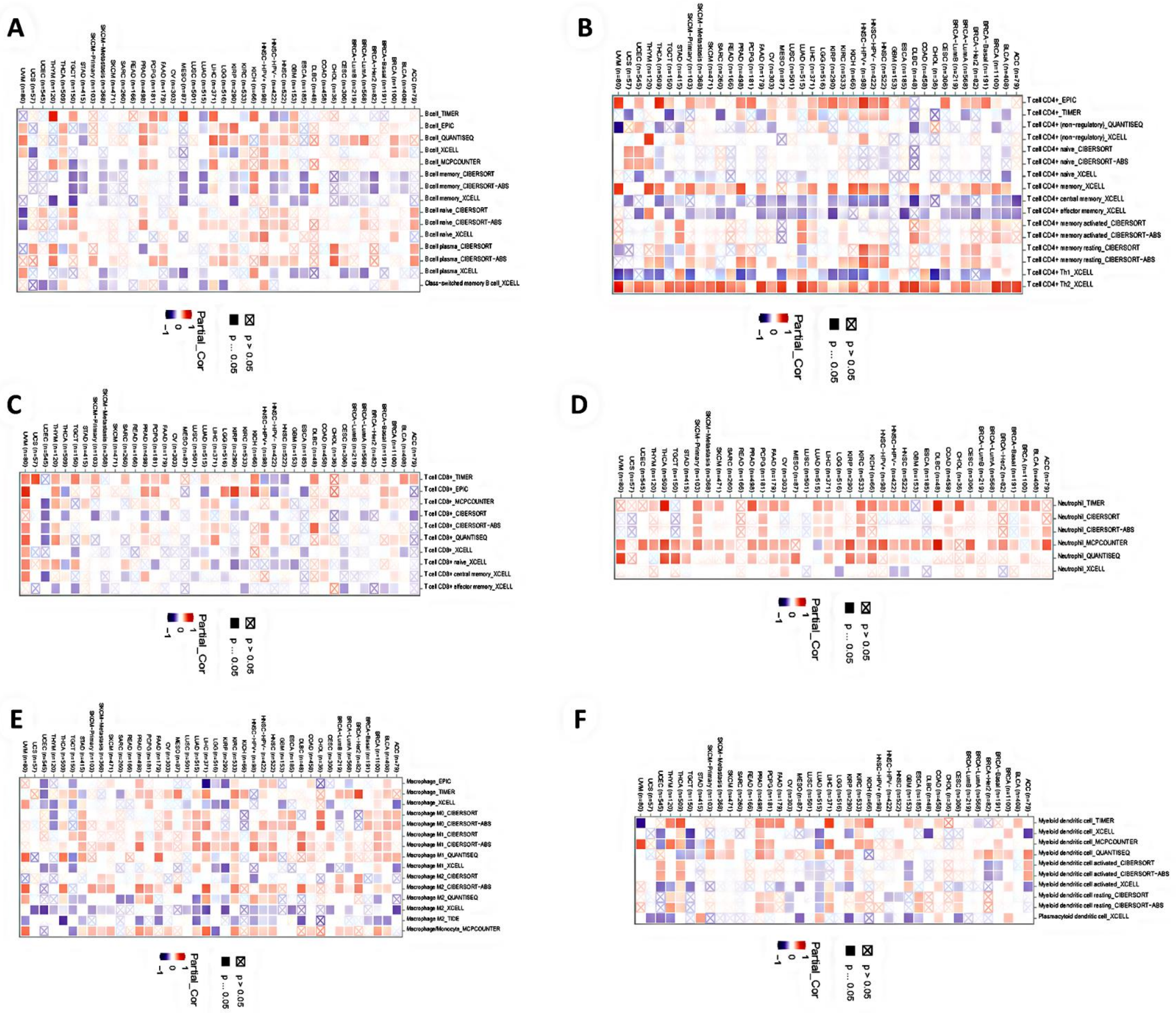

Figure S6. Correlation analysis between MCM4 expression and immune infiltration of **(A)** B cells, **(B)** CD4+ T cells, **(C)** CD8+ T cells, **(D)** neutrophils, **(E)** macrophages and **(F)** dendritic cells.

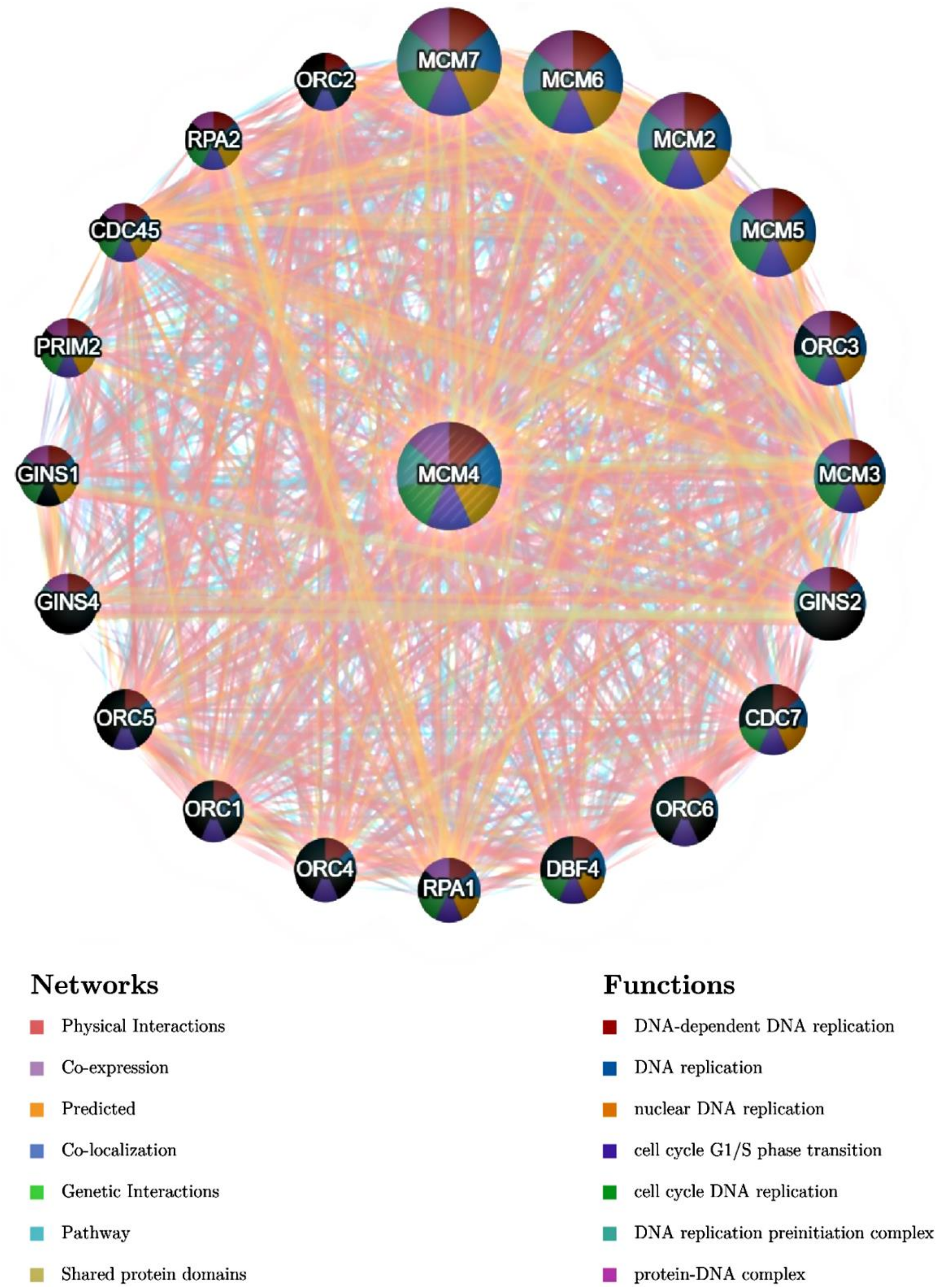

Figure S7. GGI network: 20 representative genes strongly associated with MCM4.

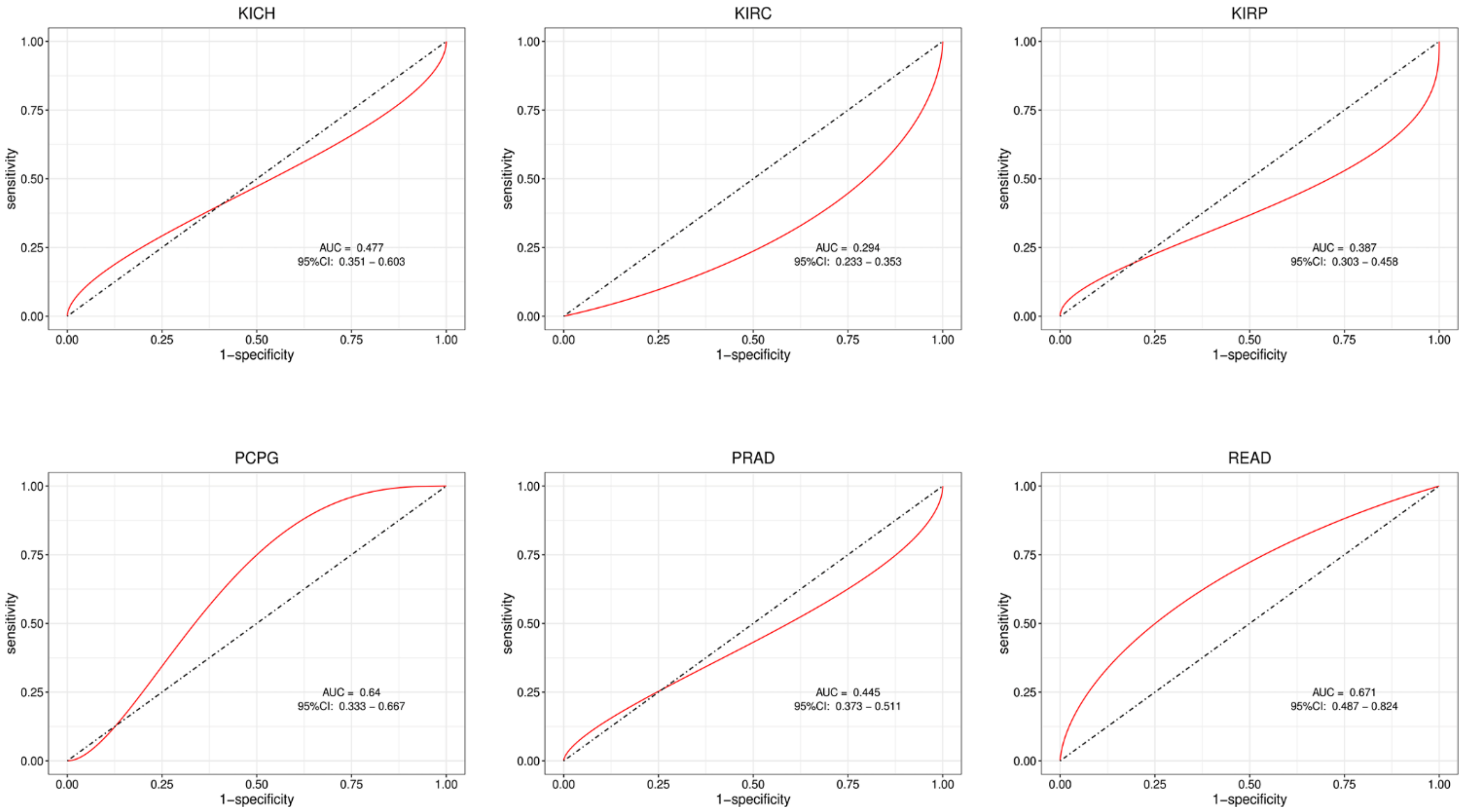

Figure S8. The ROC curve for MCM4 in KICH, KIRC, KIRP, PCPG, PRAD and READ.

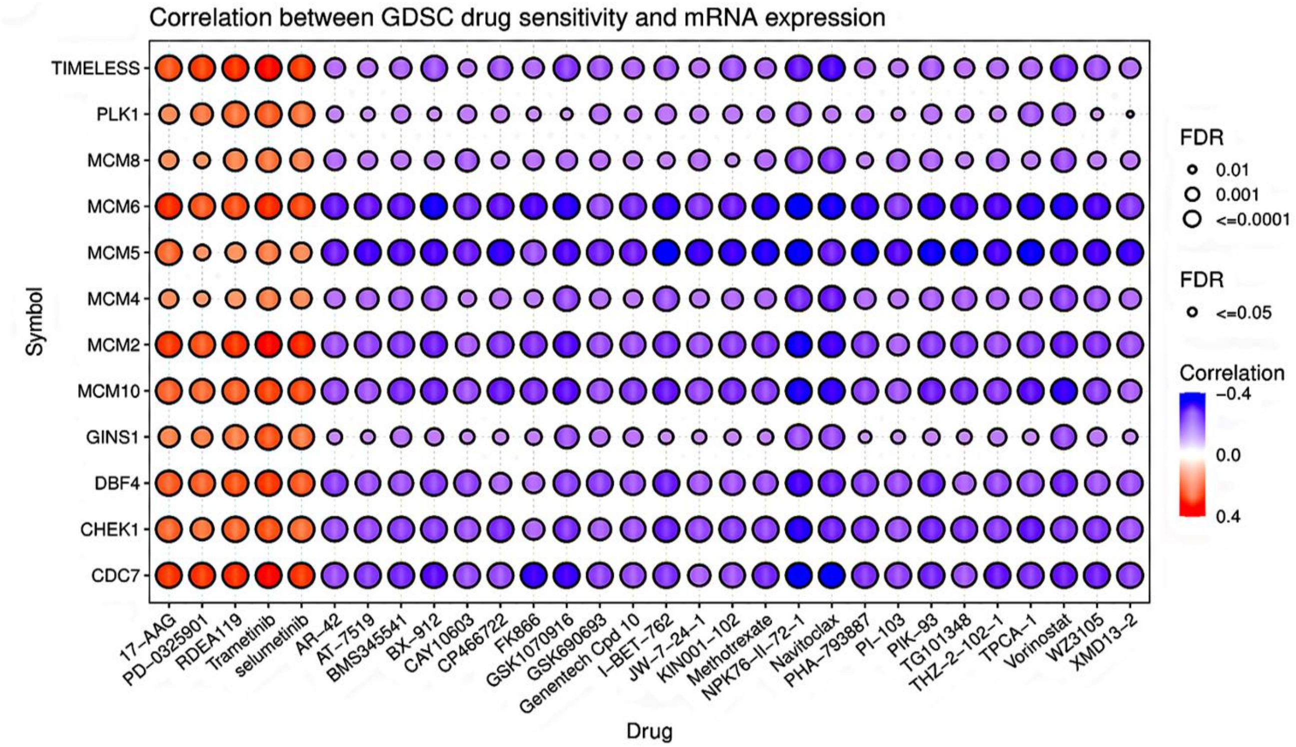

Figure S9. The relationship between the expression levels of MCM4 and genes obtained from cross analysis and different drug sensitivities.

Supplementary Tables

Table S1. Top 51 proteins in PPI network, ranked by degree method.

Proteins' Influence Rank in PPI Network		
Rank	Name	Score
1	MCM4	50
2	MCM7	44
2	MCM5	44
4	MCM6	43
5	MCM3	42
5	MCM2	42
7	MCM9	39
8	MCM8	38
9	MCMDC2	37
10	ORC1	35
11	CDC6	33
12	ORC2	32
13	CDC45	31
13	CDC7	31
15	POLA1	30
15	CDK2	30
17	CHEK2	29
17	RPA1	29
17	MCM10	29
20	CDK1	28
21	ORC3	27
22	ORC5	26
23	TIPIN	25
23	HIST1H3A	25
25	CENPA	24
26	DBF4	22
26	GINS4	22
26	HIST1H4A	22
29	RAD1	21
29	RPA2	21
29	ICK	21
29	ORC4	21
33	MAK	20
33	CCNA1	20
35	MCMBP	19
35	GINS2	19
35	SSRP1	19
35	SUPT16H	19

39	PCNA	18
39	GINS1	18
41	GINS3	17
41	CDC5L	17
41	ASF1B	17
44	ORC6	16
45	WDR18	13
46	KPNA4	9
46	POLK	9
48	SF3B1	8
49	SF3A3	4
49	DCTN2	4
51	MAPRE2	3
